# Aboveground live tree carbon stock and change in forests of conterminous United States: influence of stand age

**DOI:** 10.1186/s13021-023-00227-z

**Published:** 2023-04-16

**Authors:** Coeli M. Hoover, James E. Smith

**Affiliations:** USDA Forest Service, Northern Research Station, 271 Mast Road, Durham, NH 09824 USA

**Keywords:** Forest carbon accumulation, Aboveground live tree carbon, Stand age, Average annual change, Forest inventory and analysis

## Abstract

**Background:**

Sequestration of carbon on forest land is a common and practical component within many climate action plans developed by state or municipal governments. Initial planning often identifies the general magnitude of sequestration expected given the scope of the project. Because age plays a key role in forest carbon dynamics, we summarize both the carbon stock and accumulation rates in live trees by age class and region, allowing managers and policymakers to assess the influence of forest age class structure on forest carbon storage as represented in current inventories. State-level information is provided in supplementary tables.

**Results:**

Average regional aboveground live tree carbon stocks (represented on a per area basis) range from 11.6 tC/ha in the Great Plains to 130 tC/ha in the Pacific Northwest West (west-side of Cascades) and increase with age in all regions, although in three regions carbon stock declined in the oldest age class. Regional average annual net change in live aboveground tree carbon varies from a low of − 0.18 tC /ha/yr in the Rocky Mountain South region to a high value of 1.74 tC/ha/yr in Pacific Northwest West. In all regions except Rocky Mountain South, accumulation rates are highest in the younger age classes and decline with age, with older age classes in several western regions showing negative rates. In the Southeast and Pacific Northwest West, intermediate age classes exhibit lower rates, likely due to harvesting activity.

**Conclusions:**

Aboveground live tree carbon stocks increase and rates of average change decrease with age with few exceptions; this pattern holds when examining hardwood and softwood types individually. Because multiple forest management objectives are often considered and tradeoffs need to be assessed, we recommend considering both measures—standing stock and average annual change—of carbon storage. The relative importance of each component depends on management and policy objectives and the time frame related to those objectives. Harvesting and natural disturbance also affect forest carbon stock and change and may need to be considered if developing projections of potential carbon storage. We present forest carbon summaries at a scale and scope to meet information needs of managers and policymakers.

**Supplementary Information:**

The online version contains supplementary material available at 10.1186/s13021-023-00227-z.

## Background

While the role of forests in the global carbon cycle has been a topic of considerable attention for some time, there is increased interest in natural climate solutions for a variety of reasons, including the Trillion Trees Initiative, California’s cap and trade legislation, a growing voluntary carbon market, corporate net-zero pledges, and recent policy directives, including Executive Order 14,702 and the USDA Secretary’s Memorandum 1077-004 [1, 2]. In addition, many US states have developed (or are developing) climate action plans. Carbon sequestration is just one value of forested ecosystems, and managers, policymakers, and landowners often have multiple forest management objectives to consider, with inherent tradeoffs among the objectives. For this reason, a means to rapidly assess potential sequestration relative to other objectives can be useful to early planning. Our purpose here is to provide such information for initial scoping, by using the extensive forest inventory database compiled by the Forest Inventory and Analysis program of the USDA Forest Service.

Many variables can influence carbon stock as well as the rate of carbon accumulation, including climate, site productivity, species mix, age class distribution, stocking level, and management and disturbance history. One area of ongoing discussion is the role of older versus younger forests. Ecological theory predicts a rapid increase in ecosystem productivity, which then levels off and eventually declines in the later stages of ecological succession [3]; Ryan et al. [4] examined this phenomenon through the lens of carbon storage and proposed several explanatory mechanisms. While many studies of forested ecosystems find that the rate of carbon accumulation decreases with age [5–8], others have reported that old-growth forests continue to accumulate carbon [9, 10]. These differing results drive an ongoing discussion of the “best” strategies for managing the forest carbon sink; Repo et al. [11] provide a succinct summary of the different perspectives.

Part of the reason for seemingly conflicting results stems from the variety of quantities considered and terminology used. Some investigators, such as Luyssaert et al. [9] and Curtis and Gough [10] estimate net ecosystem productivity, which includes all pools of carbon in the ecosystem, while others focus on net primary productivity (live plant biomass) or take a carbon stock approach and quantify (different sets of) particular carbon pools; Pregitzer and Euskirchen [7] considered all three quantities. While there are accepted definitions for net ecosystem productivity and net primary productivity, Lovett et al. [12] point out that there is a trend toward redefining net ecosystem production as ecosystem carbon accumulation and they argue against such a redefinition. Terms such as carbon sequestration and carbon accumulation are often used inconsistently, and studies conducted using a stock change approach may not be directly comparable to those using flux-based methods. In addition, studies focused on forest carbon sequestration have often focused solely on carbon stocks (e.g., [13, 14]), and this may be related to the greater availability of these data relative to joint stock and change values. Nevertheless, other studies include both stocks and rates of change (e.g., [15, 8]).

While it is common for carbon stocks to be reported in the literature, a more complete picture of forest carbon sequestration consists of two components: stocks and rates of change, and these quantities may behave differently in different circumstances. Additionally, working with carbon stocks alone may make it challenging to compare different forest types, management practices, age classes, etc., since the areas under consideration may have different initial conditions. Managers may wish to place more emphasis on the rate of carbon accumulation or on the amount of carbon stock, depending on management objectives. By considering both stock and rate, managers can assess the tradeoffs among various additional management objectives not exclusively focused on carbon such as reducing wildfire risk, impact of other disturbances, or increase of ecosystem services [16, 17] as well as more traditional forest values such as timber production, wildlife habitat, and recreation.

While the exact patterns of carbon accumulation vary, forest stand development follows a relatively predicable trajectory [18]; this, combined with the legacy of significant land use change following the abandonment of agricultural land around the turn of the 20th century, shaped the United States forest carbon sink of today. As mentioned above, stand age has a large influence on forest carbon storage. To provide managers and landowners with information needed to incorporate forest carbon with other management objectives, we ask a straightforward question: how does the rate of carbon accumulation and the carbon stock in live aboveground tree biomass change with stand age in forests of the conterminous United States? We focus on live aboveground tree carbon because this is one of the largest pools, change is most easily detected, and management can have an important and rapid influence. We provide this information by summarizing forest inventory to a scale (state and small region) and scope (forest land remaining forest, expressed on a per hectare basis) to meet the information needs of state or regional forest managers and policymakers. Summarizing forest carbon as both stock and change according to broad classes of age, type, and region provides basic information in an easily accessible format for managers, landowners, or policymakers who may not have the time or resources to invest in accessing simulation models or forest inventory data retrieval tools to develop custom summaries. For example, tabular summaries can provide a means to informally analyze sensitivity of accumulation rates to alternate strategies such as slight shifts in forest type or age structure, as well as developing general estimate of expected change in carbon stocks over time.

## Materials and methods

Aboveground live tree carbon is summarized from recent forest inventory data of each of the 48 conterminous states. The forest inventory data are collected by the Forest Inventory and Analysis (FIA) Program of the USDA Forest Service. These data – the Forest Inventory and Analysis Database (FIADB) – are publicly available for download [19], and the results presented here are based on the identical 2020 inventory used in Hoover and Smith [20] to ensure consistency between summaries. We define carbon stock as the amount of carbon in live aboveground tree biomass at a point in time, expressed as carbon density (tonnes of carbon per hectare), and carbon accumulation rate (net change in carbon stock), as the difference in carbon stock in two points in time, expressed on an annual basis (tonnes per hectare per year). Methods follow Hoover and Smith [20]. Briefly, the two basic types of carbon summaries we present here—stock or accumulation -- are calculated differently to best align with the values they represent. Carbon stock densities are calculated as what are often referred to as ‘population estimates’ from the inventory data [20–22] and are based on all forest land within any particular combination of classifying attributes (e.g., stand age class, region, state, group of forest type). In contrast, carbon accumulation rates within the same classifications are based on remeasurement of permanent inventory plots with the restriction that plots remain forest land from the time-1 measurement to the time-2 measurement [20]. Our focus on accumulation within forests differs from inventory-based population estimates of change [21, 22] because the latter also include the broader effects of land use change. The carbon stock estimates are based on the most-recent inventory survey per state, and a subset of these forested plots also serve as the time-2 carbon accumulation plots when paired with time-1 from the previous survey.

**Fig. 1:**
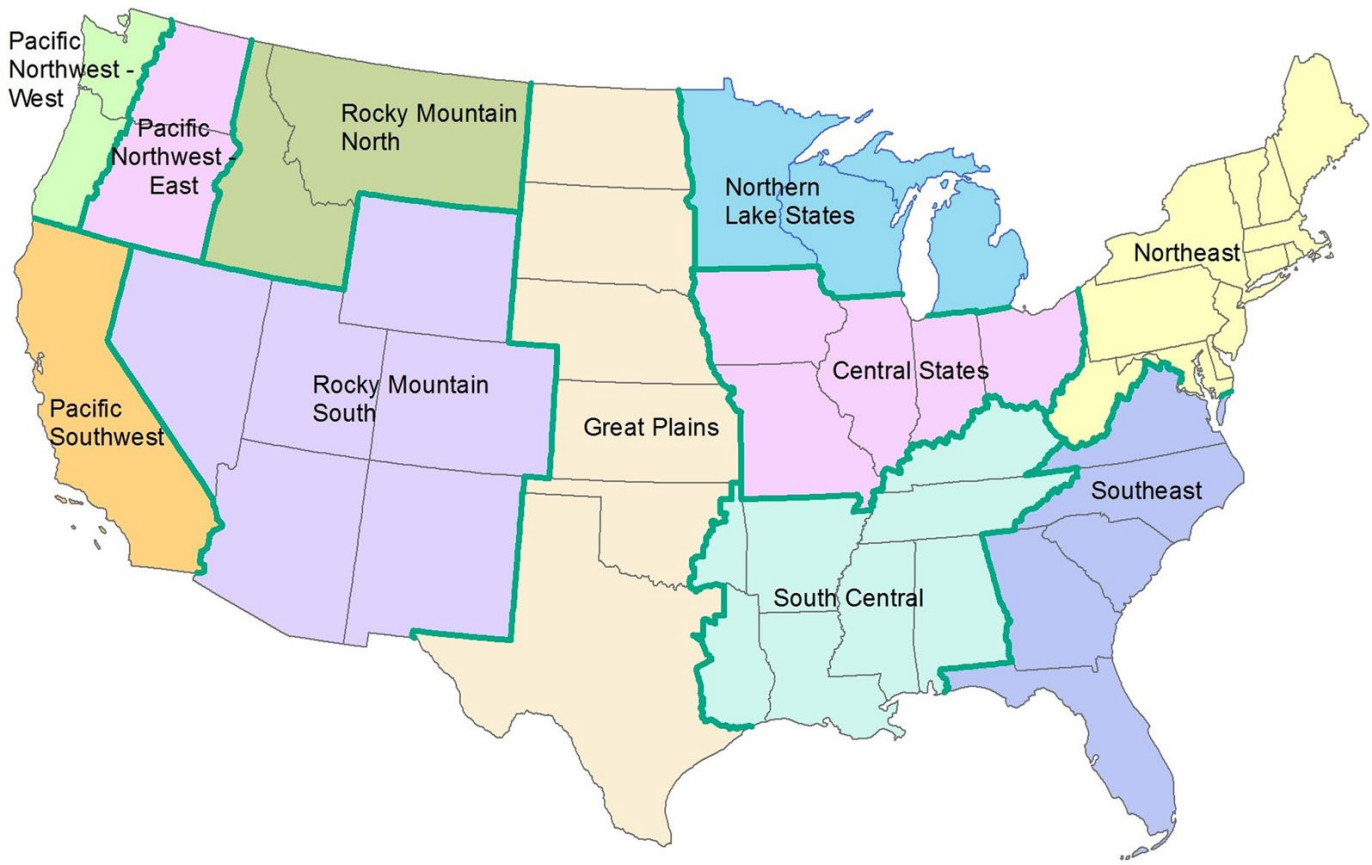
Illustration of geographic regions and component states or parts of states used for summarizing data

Classifications of forest according to region (Fig. 1), stand age, forest type, or recent disturbance are based on fields in the FIADB condition Table [22]. Age bins are according to stand age at the time-1 measurements of the remeasured pair, and age is from the FIADB [22]. Classification of recent disturbance is based on field crew observations recorded in fields of the FIADB condition table, and recent removals (or tree harvest) classification is based on records of trees cut on plots. Where the ‘no disturbance’ or ‘no removals’ labels appear in results, the only distinction from the alternate summary (i.e., ‘average’) is that we explicitly omit the respective subset of plots from the summary value [20].

General forest cover type is based on the FIADB forest type group [22], which is used to establish the three-way type classification of woodland types (typgrpcd 180,970); or softwood types (typgrpcd 100 through 390 but not 180); or hardwood types (typgrpcd 400 through 990 but not 970). Regional and state estimates are presented for hardwood, softwood, and woodland types to allow greater resolution for users who have information on the distribution of types in their area of interest; if these data are not available or forests represent combinations of groups included here, then the overall average may be more appropriate.

## Results

### Regional carbon stock and change

Overall regional average aboveground live tree carbon stock, expressed as carbon density (metric tons of carbon per hectare), ranges from a high of 130 metric tons per hectare (tC/ha) in Pacific Northwest West to a low of 11.6 tC/ha in the Great Plains region. Carbon density is a function of multiple factors including forest cover type, climate, age class distribution, and disturbance regimes. In all regions, carbon density steadily increases with age class, although in some instances carbon density declines in the oldest age class, defined as 120 + years in the East and 300 + years in the West (Table 1). In the 0–20 year age class, carbon density ranges from a low of 2.1 tC/ha in Great Plains to a high of 22.4 in South Central. Note that the youngest age class may include scattered older trees from the previous stand. In the oldest age class, carbon density varies from 22.6 tC/ha in Rocky Mountain North to 249 tC/ha in Pacific Northwest West, with most values falling between 75 and 100 tC/ha (Table 1). As mentioned above, multiple factors affect carbon density; estimates by general forest cover types (hardwood, softwood, and woodland) follow the same pattern of increasing carbon density with age, although the mean carbon density often varies among types (Additional file 1: Table S1).

**Table 1:** Carbon density (metric tons C/hectare, tC/ha) by region and age class. Estimates are for aboveground live tree carbon. SEM = standard error of the mean

Region	Age class	Mean C density (tC/ha)	SEM	Forest area (kha)	% of area (forestland)
Northeast	0–20	15.5	0.8	1,361	4
	21–40	34.8	0.6	3,513	10
	41–60	59	0.6	6,437	19
	61–80	77.1	0.5	10,445	31
	81–120	88.4	0.5	11,464	34
	121+	88.6	2.1	783	2
	Overall	70.9	0.3		
Northern Lake States	0–20	11.5	0.3	2,704	12
	21–40	29.1	0.4	3,219	15
	41–60	42	0.5	4,576	21
	61–80	52.4	0.4	5,977	27
	81–120	60.6	0.5	5,167	23
	121+	58	1.9	535	2
	Overall	43.9	0.2		
South Central	0–20	22.4	0.3	14,935	27
	21–40	54.1	0.4	13,040	23
	41–60	61.8	0.4	12,060	22
	61–80	74.3	0.5	11,994	21
	81–120	85	1	3,723	7
	121+	92.5	8.5	47	< 1
	Overall	53.7	0.2		
Southeast	0–20	22.1	0.3	10,075	28
	21–40	57.4	0.5	9,325	26
	41–60	69	0.7	5,911	16
	61–80	85.9	0.7	5,931	16
	81–120	97.4	0.9	4,525	13
	121+	99.2	3.2	295	1
	Overall	59.5	0.3		
Central States	0–20	14.1	0.9	697	5
	21–40	36.9	0.8	1,473	10
	41–60	54.4	0.6	3,881	27
	61–80	63.1	0.6	4,769	33
	81–120	70.9	0.8	3,350	23
	121+	76.7	3.6	258	2
	Overall	57.7	0.4		
Great Plains	0–20	2.1	0.1	10,789	22
	21–40	8.4	0.1	13,554	28
	41–60	14.2	0.2	14,562	30
	61–80	21.8	0.4	7,135	15
	81–120	27.2	0.8	1,930	4
	121–160	25.2	2.5	176	< 1
	161–300	37.7	8.7	33	< 1
	300+	3.4	–	6	< 1
	Overall	11.6	0.1		
Rocky Mountain-North	0–20	5.5	0.2	4,511	23
	21–40	15.5	0.5	1,231	6
	41–60	30.4	1	1,219	6
	61–80	47.4	1.4	2,016	10
	81–120	56.7	0.9	5,386	28
	121–160	58.6	1.2	2,762	14
	161–300	61.8	1.4	1,988	10
	300+	47.9	4.1	141	1
	Overall	40.2	0.4		
Rocky Mountain-South	0–20	3.8	0.1	6,456	15
	21–40	7.4	0.3	1,860	4
	41–60	10.1	0.5	1,626	4
	61–80	17.2	0.5	3,538	8
	81–120	26.8	0.4	11,268	26
	121–160	25.6	0.4	8,114	19
	161–300	23.8	0.4	9,139	21
	300+	22.6	1.4	543	1
	Overall	20.1	0.2		
Pacific Northwest - East	0–20	5.4	0.3	1,161	12
	21–40	20.7	0.6	816	8
	41–60	32.1	1.2	978	10
	61–80	41.9	1	1,743	18
	81–120	54.6	1	3,223	33
	121–160	69	2.1	1,005	10
	161–300	85.9	3.1	804	8
	300+	92	10.1	147	1
	Overall	46.1	0.6		
Pacific Northwest - West	0–20	17.3	0.8	2,032	18
	21–40	84.4	1.4	2,528	23
	41–60	135.1	2.5	1,661	15
	61–80	157.5	3.8	1,114	10
	81–120	176.7	4	1,288	12
	121–160	212.5	5.8	704	6
	161–300	234.2	4.2	1,099	10
	300+	248.9	7	610	6
	Overall	130.0	1.2		
Pacific Southwest	0–20	8.1	0.8	991	8
	21–40	49.8	2.8	689	5
	41–60	84.7	3.1	1,217	9
	61–80	77.1	2.5	1,625	13
	81–120	91.4	2	3,043	24
	121–160	110.7	3.8	1,273	10
	161–300	117.9	3.9	1,397	11
	300+	49.2	2.9	2,584	20
	Overall	76.6	1		

Overall average annual change, or the rate of carbon accumulation, varies from a low of − 0.18 tC/ha/yr (net carbon loss) in the Rocky Mountain South region to a high value of 1.74 tC/ha/yr in Pacific Northwest West, with considerable variability among the regions (Table 2). As with carbon density, accumulation rates are a function of multiple factors, and vary across the age classes, with rates generally declining as age increases. In the youngest age class, accumulation rates range from a high of 3 tC/ha/yr in Pacific Northwest West to a low of 0.03 tC/ha/yr in Rocky Mountain South, with most rates between 1 and 2 tC/ha/yr. In the oldest age class, accumulation is highest in South Central at 1.33 tC/ha/yr, although the number of plots is low; the next highest value is 0.52 tC/ha/yr in the Southeast. In the oldest age class the lowest rate of -1.46 tC/ha/yr is found in the Great Plains region, although sample size is small; negative rates are present in five regions (Table 2). Carbon accumulation rates by general forest cover type are presented where at least 30 pairs of remeasured plots are available (Additional file 2: Table S2); rates show the same pattern of decrease from younger to older age classes. Where rate data are available for both hardwood and softwood types, accumulation rates are higher in the softwoods; however, in the Northeast, South Central, and Southeast regions this applies only to the younger age classes. Forested area by age class and type is provided in (Additional file 3: Table S3) for those wishing to use this information to develop generalized carbon stock or change estimates.

**Table 2:** Carbon accumulation rates (metric tons C/hectare/year, tC/ha/yr) by region and age class. Estimates are for aboveground live tree carbon. N = number of paired plots on which the estimate is based

Region	Age class	Average rate (tC/ha/yr)	N
Northeast	0–20	1.47	425
	21–40	1.16	897
	41–60	0.69	1823
	61–80	0.36	2782
	81–120	0.27	2083
	120+	0.07	136
	Overall	0.55	
Northern Lake States	0–20	1.13	946
	21–40	0.82	1221
	41–60	0.26	2008
	61–80	0.14	2392
	81–120	0.16	1343
	120+	0.41	184
	Overall	0.40	
South Central	0–20	2.41	2536
	21–40	0.52	1780
	41–60	0.37	2614
	61–80	0.11	1791
	81–120	0.04	433
	120+	1.33	7
	Overall	0.90	
Southeast	0–20	2.03	2422
	21–40	0.29	1641
	41–60	0.49	1354
	61–80	0.45	1335
	81–120	0.77	763
	120+	0.57	61
	Overall	0.96	
Central States	0–20	1.17	152
	21–40	0.97	490
	41–60	0.39	1058
	61–80	0.18	1025
	81–120	0.07	551
	120+	−0.49	47
	Overall	0.38	
Great Plains	0–20	0.28	12
	21–40	0.69	81
	41–60	0.38	115
	61–80	−0.14	107
	81–120	−0.39	91
	121–160	−1.46	13
	161–300	0.17	4
	300+	–	
	Overall	0.08	
Rocky Mountain-North	0–20	0.54	421
	21–40	0.85	163
	41–60	0.78	192
	61–80	0.52	444
	81–120	−0.25	956
	121–160	−0.63	470
	161–300	−1.00	411
	300+	−0.80	19
	Overall	−0.70	
Rocky Mountain-South	0–20	0.03	586
	21–40	0.02	229
	41–60	0.10	339
	61–80	0.03	776
	81–120	−0.19	2294
	121–160	−0.30	1578
	161–300	−0.28	1883
	300+	−0.14	112
	Overall	−0.18	
Pacific Northwest - East	0–20	0.71	251
	21–40	1.09	239
	41–60	0.75	446
	61–80	0.44	890
	81–120	0.26	1167
	121–160	0.35	411
	161–300	0.28	406
	300+	0.42	42
	Overall	0.45	
Pacific Northwest - West	0–20	3.06	516
	21–40	3.45	626
	41–60	0.33	355
	61–80	1.03	339
	81–120	1.63	449
	121–160	1.50	290
	161–300	0.64	555
	300+	0.38	205
	Overall	1.74	
Pacific Southwest	0–20	1.32	94
	21–40	2.50	161
	41–60	1.17	268
	61–80	0.90	437
	81–120	0.45	650
	121–160	0.07	293
	161–300	−0.15	368
	300+	−0.64	73
	Overall	0.58	

### State level carbon stocks and change

Live tree aboveground carbon varies considerably by state, although the pattern of increasing carbon density with age class is consistent (Additional file 4: Table S4). As with the regional estimates, the exception is the very oldest age class, where carbon density may decline (occasionally no plots representing the oldest age class are present in the database). Estimates are presented by hardwood/softwood/woodland types for those users who wish to develop estimates for particular states (Additional file 4: Table S4). Carbon density in woodland types (when present) is always lower than that in hardwood or softwood types regardless of age class, as expected due to growth form. However, in most states the carbon density in hardwood and softwood types is generally similar; some exceptions include Florida, Kansas, Oregon, Nebraska, the portion of Washington in the Pacific Northwest West region, and West Virginia. Carbon accumulation rates by age class and type (Additional file 5: Table S5) are estimated in those cases where at least 30 remeasured pairs are available; note that error of any estimate decreases as sample size increases. In multiple instances, estimates of carbon stocks, but not accumulation rates, are available for a particular age class/forest cover type combination. Again, the pattern of declining accumulation rate with increasing age is apparent for softwood and hardwood forest types at the state level; this is less apparent for the relatively sparse number of woodland types. Forested area by the classifications in (Additional files 4 and 5: Tables S4 and S5) are provided in (Additional file 6: Table S6).

## Discussion

Our primary objective is to provide information regarding the effect of forest age class on current and potential future carbon storage, in terms of both stocks and rates. Classification by age has a record of being an important feature in assessing forest carbon accumulation (e.g., [23, 24]). In addition to our use of age bins to summarize how carbon stocks and accumulation rates change from younger- to older-age stands, they also provide a quick overview of how stand age is distributed among current forest lands. Note that, while the 20-year stand age bins are convenient for these summaries, they can include a wide range of stand conditions, especially in younger stands where scattered older trees from the prior stand may remain. Stand ages provided in the FIA database are based on a few representative trees [22], and a harvest or disturbance that removes a portion of the trees will have the subsequent calculated stand age based on a new set of representative trees. This is one of the reasons for including the ‘no removals’ and ‘no disturbance’ classifications.

While we present stock (tC/ha) and accumulation (tC/ha/y) in terms of carbon density, managers may also need to estimate total carbon, which is computed as the product of average density and total forest area (areas are supplied in Table 1 (Additional file 3: Table S3) and  (Additional file 6: Table S6). Because the rate of carbon accumulation (with a few exceptions) is higher in younger stands, (Table 2) the distribution of forest area by age class has important implications for future carbon storage. Trends in the distribution of these age classes vary within regions; in Southeast, South Central, and Great Plains, around 50% of forestland is classified as 40 years or younger, while in Northeast, Central States, Rocky Mountain South, Pacific Northwest East, and Pacific Southwest this age class represents less than 20% of forested area (Table 1).

In most regions and states, the highest rates of carbon accumulation are found in the early and mid-stages of forest stand development (Table 2, Additional file 2: Table S2), as expected. Gray et al. [25] investigated carbon stock and accumulation in Pacific Northwest forests and found that accumulation rates varied significantly with age, with higher rates often found in younger stands, but also noted that site productivity class and plant community type had important effects on patterns of carbon accumulation. Lu et al. [6] conducted regional and state level analyses of carbon sequestration for the conterminous United States using the Terrestrial Ecosystem Model and found a strong negative correlation between stand age and the rate of carbon accumulation per unit of forest area. Using a carbon cycle model to examine carbon sequestration in the Southeast United States, Gu et al. [26] also found stronger carbon sinks in portions of the study area where forestland was dominated by younger and near-mature pine stands and weaker sink strength in oak-hickory stands in mid-to-late successional stages. Gough et al. [27], Coulston et al. [5], and Jonsson et al. [17], among others, also report decreasing carbon accumulation rates with increasing stand age. While Luyssaert et al. [9] concluded that old-growth forests continued to accumulate carbon (based on net ecosystem productivity), they also noted that when net primary productivity of temperate and boreal forests is analyzed separately, rates decline over time.

**Fig. 2:**
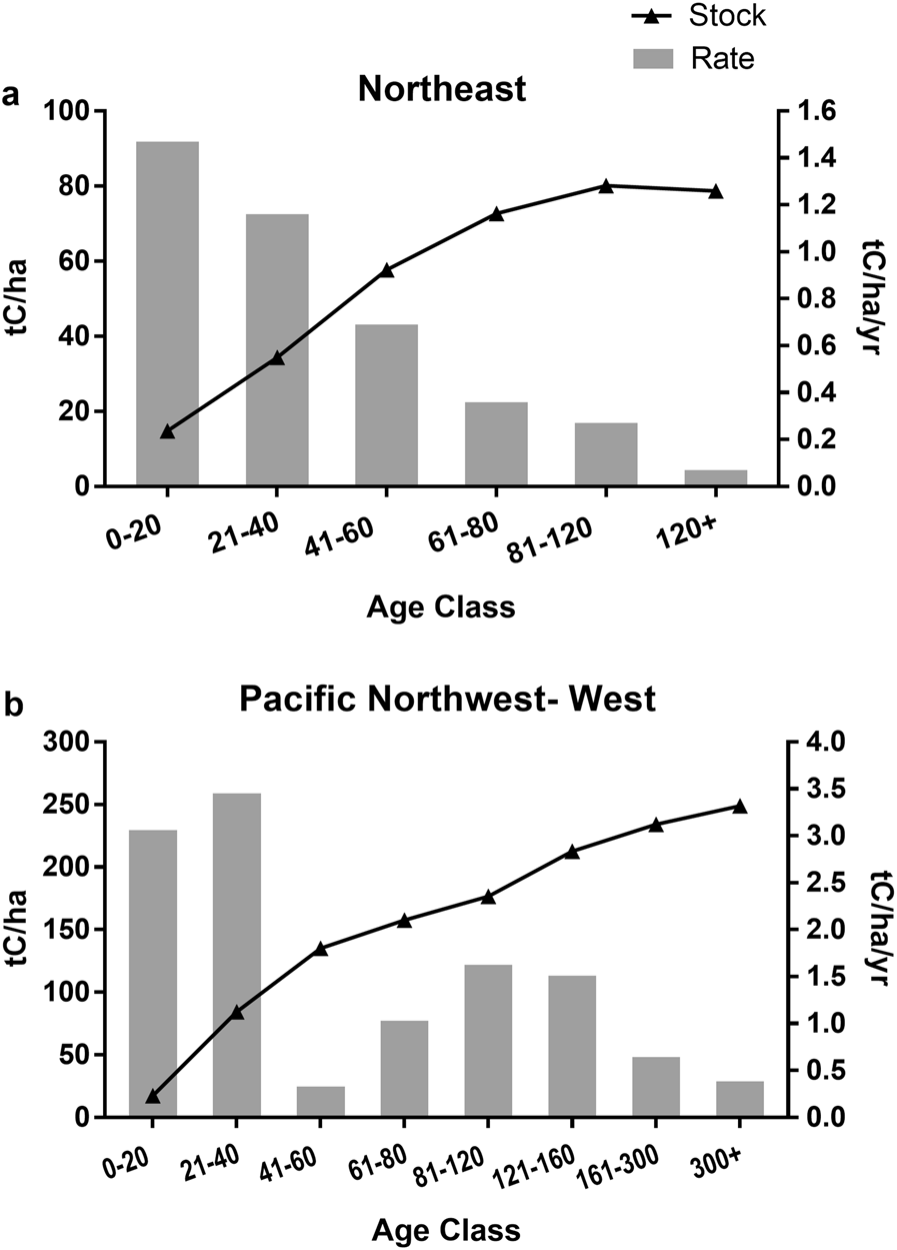
Average stocks (tC/ha, line) and annual net change (tC/ha/yr, bars) in live aboveground tree carbon stocks by age class in the **a** Northeast and **b** Pacific Northwest – West regions

**Fig. 3:**
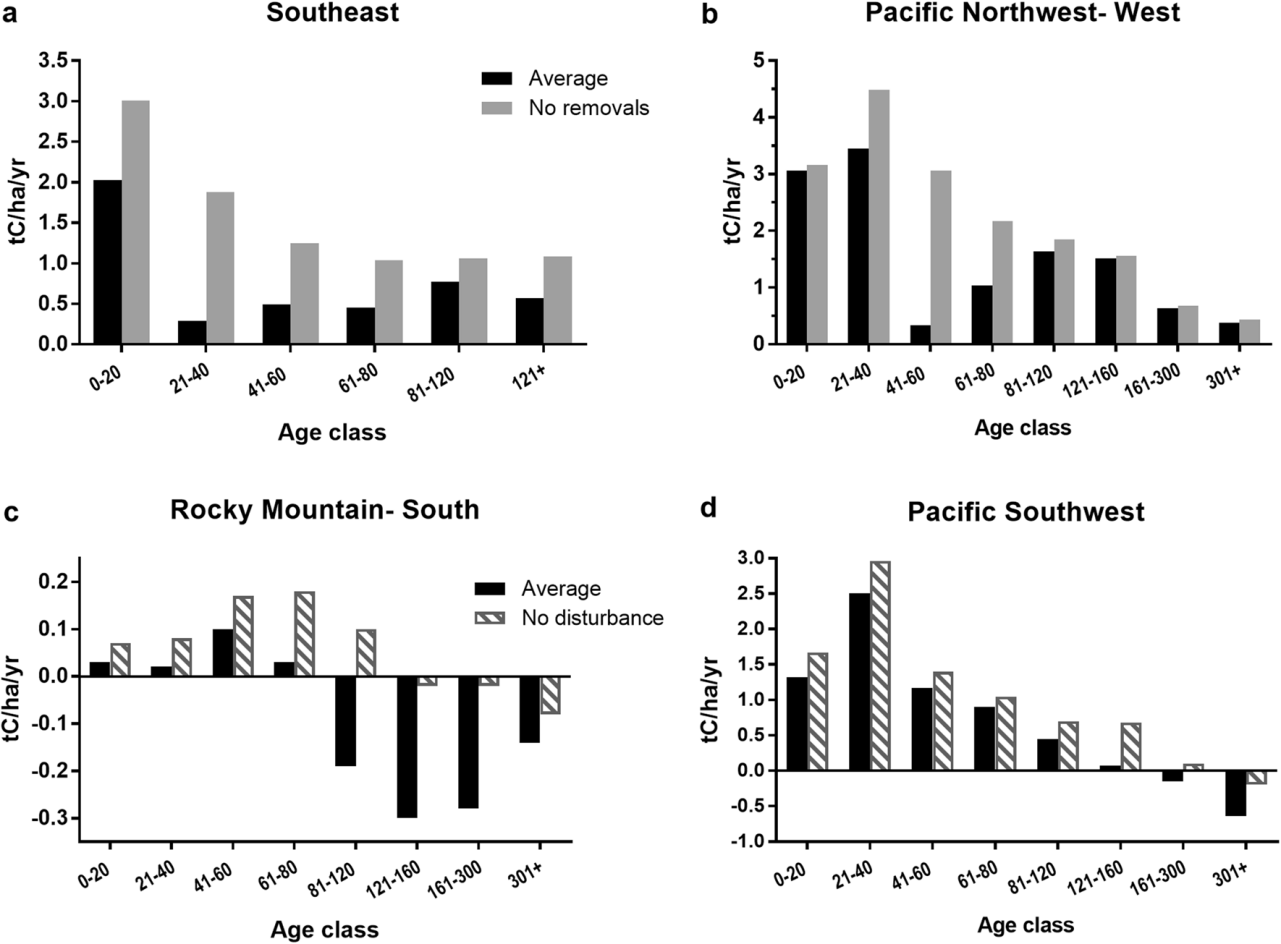
Average annual change in live aboveground tree carbon stocks (tC/ha/yr) by age class with effect of removing plots with identified removals (harvest) from regional averages in **a** Southeast or **b** Pacific Northwest – West or removing plots with identified disturbances from regional averages in **c** Rocky Mountain – South or **d** Pacific Northwest – West regions

The pattern of increasing carbon stocks with stand age (Table 1, Additional file 1: Table S1 and Additional file 4: Table S4), followed by a leveling off or slight decline, is as expected and widely reported (e.g., [28, 17, 29]). Most regions in the United States follow the pattern of increasing stock (with occasional declines in the oldest age classes) and decreasing rate as stand age increases (Fig. 2a), a pattern commonly seen in studies reporting both stocks and rates [8, 11, 15, 30]. Note that Bradford et al. [31] report that tree biomass increment and net primary productivity did not decline in older stands, which may be due to the presence of mixed-aged stands. In a few regions (notably Southeast and Pacific Northwest West), the pattern was different: while stock increased over time, the rate showed a sharp decrease between 20 and 40 years, followed by an increase and then a gradual decline (Fig. 2b). A possible explanation is harvesting or natural disturbance; Gray et al. [32], Gu et al. [26], and Harris et al. [33] estimated the effects of harvest and natural disturbance on United States forest carbon stocks. Effects varied by region, with largest impacts generally in the South and West; Williams et al. [16] also discuss the role of disturbance in forest carbon dynamics. Records in the FIA database include information on harvest and other disturbance and we use those fields to examine the possible impact on our estimates. When plots with harvesting activity during or immediately prior to our study period are excluded, aboveground live tree carbon accumulation rates increase considerably in these regions, particularly in the 21–40 (Southeast) and 41–60 (Pacific Northwest West) age classes (Fig. 3a and b). Carbon accumulation rates are negative in the older age classes in several western regions, most notably in the Rocky Mountains (Table 2); when plots with a recent history of natural disturbance are excluded the effect is noticeable, indicating that disturbance is the likely driver of the decline in rate (Fig. 3c and d). Note that carbon in trees removed from harvesting or killed as a result of natural disturbance is not immediately released to the atmosphere (even in the case of wildfire, some material remains) but transferred to other carbon pools (such as standing dead trees) and is then released over time as decomposition occurs.

Landowners and managers evaluate multiple factors and tradeoffs among objectives when developing a management plan. As mentioned previously, there are two components of forest carbon dynamics: stock and the rate of change. Both are important considerations; management action may not affect both components in the same manner, or one aspect may have a higher priority based on overall management objectives (which may include wildlife habitat and sustainable timber production, for example). In some forest types and regions where stands may be overstocked and at higher risk of wildfire, drought, or disease, maximizing carbon stocks may not be an optimal practice. Considering stocks and rates in the context of landscape-level management objectives allows managers to assess tradeoffs and develop management plans that optimize results across the landscape, rather than focus on maximizing a particular element. As others have noted, it is not possible to simultaneously maximize stocks and rates in a stand; D’Amato et al. [15] provide a concise overview of the tradeoffs between managing for climate mitigation and adaptation. In many regions and states outside the South, the age class distribution of forest stands is skewed toward older age classes (Table 1, Additional file 6: Table S6), which may result in a high carbon density but a slowing rate of carbon accumulation and a weaker sink in the future. These tradeoffs apply to other management objectives as well; for example, in the Northeast a small proportion of forested area is in the younger age classes (Table 1), resulting in a lack of the early successional habitat needed by many mammal and bird species [34, 35].

The age-class specific stock and accumulation rates provided here, combined with the distribution of forested area by age class, enables mangers and landowners to assess the carbon implications of potential management actions and evaluate tradeoffs among management objectives. Disturbance history, site productivity, stocking level, management history, and other variables all affect forest carbon dynamics; however, stand age has consistently been shown to be a key driver of forest carbon stocks and accumulation. When including forest carbon as a management objective, considering both stock and accumulation rate provides a more comprehensive picture of forest carbon dynamics and facilitates planning to optimize outcomes among multiple objectives.

## Conclusion

The goal of this study is to examine the influence of stand age on both carbon stock density and accumulation rate in forests of the conterminous United States, and to provide convenient summaries of forest carbon data for managers, landowners, and policymakers. With the increased focus on natural climate solutions and the need to evaluate tradeoffs between forest carbon and more traditional management objectives, easily accessible information on stocks and rates is an important resource to inform management plan development and assess the future potential of the forest carbon sink. In each region of the United States, the rate of carbon accumulation is highest in youngest age classes (0–20 years) and declines with age; harvesting activity resulted in notable drops in the accumulation rate in some intermediate age classes in the Southeast (21–40 year) and Pacific Northwest West (41–60 year) regions. In some regions, accumulation rates are negative in the older age classes, which in some cases is related to disturbance. Carbon stock (per hectare) increased with age in all regions, although in some instances the oldest age class showed a slight decline.

The pattern of increasing stocks and decreasing rates with age has implications for the future forest carbon sink; managers developing forest carbon projects or plans may wish to consider the age-class distribution of forested lands during the analysis and planning process. Since it is not possible to maximize both stock and rate, we recommend considering both components of carbon dynamics; the relative weight given to each will depend on management and policy objectives. Finally, harvesting and natural disturbance also affect the forest carbon sink and may need to be included when developing projections of future carbon storage potential or plans related to maintaining or enhancing the forest carbon sink.

### Supplementary Information

**Additional file 1: Table S1. **Aboveground live tree carbon density by region and age class (metric tons C/hectare, tC/ha) grouped by hardwood and softwood types.  SEM = standard error of the mean. Values less than 0.1 are displayed as zeroes; empty cells indicate no data for that category.

**Additional file 2: Table S2. **Regional carbon accumulation rates (metric tons C/hectare/year, tC/ha/yr) grouped by hardwood, softwood, and woodland  types. Estimates are for aboveground live tree carbon.  N = number of paired plots on which the estimate is based; data are shown only if N ≥ 30.  Note that the error of the estimate decreases with increasing N.

**Additional file 3: Table S3. **Forested area by region, type (softwood, hardwood, woodland), and age class (kha, thousand hectares).  % = percentage of total forestland in that age class. Values less than one percent are displayed as zeroes; empty cells indicate no data for that category.

**Additional file 4: Table S4. **Mean carbon density (metric tons C/hectare,  tC/ha) by state, type (softwood, hardwood, woodland) and age class.  Estimates are for aboveground live tree carbon. SEM = standard error of the mean. For states that span more than one region, carbon density is given for the entire state as well as the portion in each region. Values less than one percent are displayed as zeroes; empty cells indicate no data for that category.

**Additional file 5: Table S5. **Carbon accumulation rates (metric tons C/hectare/year, tC/ha/yr) by state, type (softwood, hardwood, woodland) and age class.  Estimates are for aboveground live tree carbon. N = number of paired plots on which the estimate is based; data are shown only if N ≥ 30.  Categories are omitted if no bins meet the N≥ 30 cutoff; Delaware, Nebraska, North Dakota, Rhode Island, Wyoming, and the Great Plains portions of Oklahoma and Texas are not represented in this table because no categories met the sample size threshold.  Note that the error of the estimate decreases with increasing N. For states that span more than one region, rates are given for the entire state as well as the portion in each region (if sufficient data are available).

**Additional file 6: Table S6. **Forested area by state, type (softwood, hardwood, woodland) and age class (kha, thousand hectares).  % = percentage of total forestland in that age class. Values less than one percent are displayed as zeroes.  Note that areas may not sum to total; “All” includes nonstocked forestland, while types include only forestland classified as stocked.  Values are also rounded. For states that span two regions, areas are given for the entire state and the portion in each region. Values less than one percent are displayed as zeroes; empty cells indicate no data for that category.

## Data Availability

Data are publicly available at https://apps.fs.usda.gov/fia/datamart/datamart.html. Note that the FIA Database is updated as new data are added, and as a result, will be slightly different from the dataset used here.
