# Correction to: Aboveground live tree carbon stock and change in forests of conterminous United States: influence of stand age

**DOI:** 10.1186/s13021-024-00265-1

**Published:** 2024-07-13

**Authors:** Coeli M. Hoover, James E. Smith

**Affiliations:** USDA Forest Service, Northern Research Station, 271 Mast Road, Durham, NH 09824 USA

**Correction to: Carbon Balance and Management (2023) 18:7**

10.1186/s13021-023-00227-z

Following publication of the original article [1], the authors identified the errors in the supplementary materials. The table captions and some incorrect values in Table S6 were updated. The corrected supplementary tables have been uploaded with this correction.

The original article has been corrected.

### Electronic supplementary material

Below is the link to the electronic supplementary material.

**Additional file 1: Table S1.** Aboveground live tree carbon density by region and age class (metric tons C/hectare, tC/ha) grouped by hardwood and softwood types. SEM = standard error of the mean. Values less than 0.1 are displayed as zeroes; empty cells indicate no data for that category.

**Additional file 2: Table S2.** Regional carbon accumulation rates (metric tons C/hectare/year, tC/ha/yr) grouped by hardwood, softwood, and woodland types. Estimates are for aboveground live tree carbon. N = number of paired plots on which the estimate is based; data are shown only if N ≥ 30. Note that the error of the estimate decreases with increasing N.

**Additional file 3: Table S3.** Forested area by region, type (softwood, hardwood, woodland), and age class (kha, thousand hectares). % = percentage of total forestland in that age class. Values less than one percent are displayed as zeroes; empty cells indicate no data for that category.

**Additional file 4: Table S4.** Mean carbon density (metric tons C/hectare, tC/ha) by state, type (softwood, hardwood, woodland) and age class. Estimates are for aboveground live tree carbon. SEM = standard error of the mean. For states that span more than one region, carbon density is given for the entire state as well as the portion in each region. Values less than one percent are displayed as zeroes; empty cells indicate no data for that category.

**Additional file 5: Table S5.** Carbon accumulation rates (metric tons C/ hectare/year, tC/ha/yr) by state, type (softwood, hardwood, woodland) and age class. Estimates are for aboveground live tree carbon. N = number of paired plots on which the estimate is based; data are shown only if N ≥ 30. Categories are omitted if no bins meet the N ≥ 30 cutoff; Delaware, Nebraska, North Dakota, Rhode Island, Wyoming, and the Great Plains portions of Oklahoma and Texas are not represented in this table because no categories met the sample size threshold. Note that the error of the estimate decreases with increasing N. For states that span more than one region, rates are given for the entire state as well as the portion in each region (if sufficient data are available).

**Additional file 6: Table S6.** Forested area by state, type (softwood, hardwood, woodland) and age class (kha, thousand hectares). % = percentage of total forestland in that age class. Values less than one percent are displayed as zeroes. Note that areas may not sum to total; “All” includes nonstocked forestland, while types include only forestland classified as stocked. Values are also rounded. For states that span two regions, areas are given for the entire state and the portion in each region. Values less than one percent are displayed as zeroes; empty cells indicate no data for that category.

## References

[1] Hoover CM, Smith JE (2023). Aboveground live tree carbon stock and change in forests of conterminous United States: influence of stand age. Carbon Balance Manage.

