# Supplementary material for: Aboveground live tree carbon stock and change in forests of conterminous United States: influence of stand age
Source: Carbon Balance Manag. 2023 Apr 16;18:7. doi: 10.1186/s13021-023-00227-z (PMC10108445; doi:10.1186/s13021-023-00227-z)
Supplement: Supplementary file 1 — Additional file 1: Table S1. Aboveground live tree carbon density by region and age class (metric tons C/hectare, tC/ha) grouped by hardwood and softwood types. SEM = standard error of the mean. Values less than 0.1 are displayed as zeroes; empty cells indicate no data for that category. [file 13021_2023_227_MOESM1_ESM.pdf]

Table S1 . Aboveground live tree carbon density by region and age class (metric tons C/hectare, tC/ha) grouped by hardwood and softwood types. SEM = standard error of the mean. Values less than 0.1 are displayed as zeroes; empty cells indicate no data for that category.

Region	Group		0-20	21-40	41-60	61-80	81-120	121+	121-160	161-300	301+
Northeast	SW	tC/ha	17.8	35.0	51.0	65.1	69.9	68.2			
		SEM	1.4	0.9	1.3	1.3	1.3	3.1			
	HW	tC/ha	17.5	34.7	60.5	79.1	91.3	97.8			
		SEM	1.1	0.7	0.6	0.5	0.5	2.4			
Northern Lake States	SW	tC/ha	11.0	28.0	36.5	39.9	44.1	49.3			
		SEM	0.8	0.8	0.8	0.9	0.9	2.2			
	HW	tC/ha	12.6	29.5	44.0	55.9	66.0	72.6			
		SEM	0.3	0.5	0.5	0.5	0.6	3.0			
South Central	SW	tC/ha	28.7	63.9	74.7	83.1	88.3				
		SEM	0.4	0.5	1.0	1.3	3.1				
	HW	tC/ha	16.2	43.0	58.4	73.1	84.7	92.5			
		SEM	0.3	0.5	0.4	0.5	1.0	8.5			
	WL	tC/ha	3.1	6.9	16.2	34.4	49.4				
		SEM	0.2			5.6	1.4				
Southeast	SW	tC/ha	28.3	63.3	70.9	80.8	81.9	56.7			
		SEM	0.5	0.6	1.3	2.0	3.0	16.1			
	HW	tC/ha	17.1	48.8	68.1	87.0	98.9	101.3			
		SEM	0.4	0.7	0.8	0.8	1.0	3.1			
Central States	SW	tC/ha	18.1	30.6	48.5	59.7	60.5	86.6			
		SEM	4.0	4.0	2.9	3.7	6.4				
	HW	tC/ha	15.8	37.3	54.6	63.1	71.0	76.6			
		SEM	1.0	0.9	0.6	0.6	0.8	3.6			

Region	Group		0-20	21-40	41-60	61-80	81-120	121+	121-160	161-300	301+
Great Plains	SW	tC/ha	6.3	14.8	20.9	24.9	30.5		32.2	42.6	
		SEM	0.9	1.2	1.3	2.2	1.4		3.1	7.5	
	HW	tC/ha	6.7	17.4	23.5	30.0	33.2		24.1	36.1	
		SEM	0.4	0.4	0.4	0.6	1.2		3.6	11.5	
	WL	tC/ha	1.4	4.5	7.1	11.2	13.6		16.6		3.4
		SEM	0.1	0.1	0.1	0.3	0.8		6.4		
Rocky Mountain- North	SW	tC/ha	8.1	16.2	33.5	52.1	59.3		60.8	63.7	48.7
		SEM	0.3	0.6	1.1	1.5	0.9		1.2	1.4	4.1
	HW	tC/ha	6.2	15.9	24.5	27.8	39.0		28.7	64.6	
		SEM	0.7	2.4	2.7	2.8	3.7		6.1	26.5	
	WL	tC/ha	3.9	5.3	6.2	7.6	11.6		11.0	12.7	0
		SEM	1.1	0.8	0.8	0.7	1.0		1.1	1.5	
Rocky Mountain-South	SW	tC/ha	8.8	11.8	19.9	30.9	43.0		50.4	56.0	61.5
		SEM	0.5	0.6	1.3	0.9	0.6		1.0	1.3	8.5
	HW	tC/ha	7.5	13.2	25.7	31.6	46.1		55.8	44.4	
		SEM	0.5	1.1	2.0	1.5	1.4		3.4	12.8	
	WL	tC/ha	3.8	4.6	5.3	8.2	10.9		13.0	15.9	19.1
		SEM	0.2	0.3	0.2	0.3	0.2		0.2	0.2	1.1
Pacific Northwest - East	SW	tC/ha	9.3	20.7	32.1	42.2	55.4		69.7	86.2	105.6
		SEM	0.6	0.6	1.2	1.1	1.0		2.1	3.1	11.5
	HW	tC/ha	3.2	19.5	32.3	35.7	34.7		27.3	11.8	30.2
		SEM	1.2	3.9	13.5	6.2	3.7		9.1	1.3	7.4
	WL	tC/ha		0.5	8.7		4.8		13.0		
		SEM		0.2	5.6		3.1				
Pacific Northwest - West	SW	tC/ha	19.5	87.7	150.1	168.3	189.8		218.3	235.7	265.8
		SEM	1.0	1.5	2.8	4.5	4.4		5.9	4.3	6.7
	HW	tC/ha	17.6	66.6	86.8	124.0	115.2		121.6	169.3	56.3
		SEM	1.7	3.0	3.6	5.6	6.4		21.7	19.1	9.6

Region	Group		0-20	21-40	41-60	61-80	81-120	121+	121-160	161-300	301+
Pacific Southwest	SW	tC/ha	14.4	50.5	83.9	78.0	94.4		120.1	127.5	157.0
		SEM	2.0	3.5	4.4	3.0	2.4		4.3	4.4	20.4
	HW	tC/ha	10.3	49.4	85.7	79.1	88.5		96.4	99.4	41.3
		SEM	1.6	4.7	4.3	4.6	4.3		8.7	8.8	1.4
	WL	tC/ha	0.3	2.0	1.5	12.7	11.2		7.7	16.8	8.6
		SEM	0.2	0.6	0.0	3.3	1.9		1.6	1.9	0.7
