# Supplementary material for: Aboveground live tree carbon stock and change in forests of conterminous United States: influence of stand age
Source: Carbon Balance Manag. 2023 Apr 16;18:7. doi: 10.1186/s13021-023-00227-z (PMC10108445; doi:10.1186/s13021-023-00227-z)
Supplement: Supplementary file 2 — Additional file 2: Table S2. Regional carbon accumulation rates (metric tons C/hectare/year, tC/ha/yr) grouped by hardwood, softwood, and woodland types. Estimates are for aboveground live tree carbon. N = number of paired plots on which the estimate is based; data are shown only if N ≥ 30. Note that the error of the estimate decreases with increasing N. [file 13021_2023_227_MOESM2_ESM.pdf]

Table S2. Regional carbon accumulation rates (metric tons C/hectare/year, tC/ha/yr) grouped by hardwood, softwood, and woodland types. Estimates are for aboveground live tree carbon. N = number of paired plots on which the estimate is based; data are shown only if N ≥ 30. Note that the error of the estimate decreases with increasing N.

Region	Group		0-20	21-40	41-60	61-80	81-120	121+	121-160	161-300	301+
Northeast	SW	tC/ha/yr	1.78	1.35	0.58	0.40	0.16	0.25			
		N	158	271	330	388	350	50			
	HW	tC/ha/yr	1.29	1.08	0.72	0.35	0.29	-0.04			
		N	267	626	1493	2394	1733	86			
Northern Lake States	SW	tC/ha/yr	1.22	0.80	0.39	0.33	0.23	0.38			
		N	210	362	513	503	435	127			
	HW	tC/ha/yr	1.10	0.82	0.21	0.09	0.13	0.47			
		N	736	859	1495	1889	908	57			
South Central	SW	tC/ha/yr	2.84	0.54	0.01	-0.63	-0.55				
		N	1739	760	506	240	38				
	HW	tC/ha/yr	1.48	0.50	0.46	0.23	0.10				
		N	797	1020	2108	1551	394				
Southeast	SW	tC/ha/yr	2.51	0.26	0.11	0.08	-0.69				
		N	1600	889	341	190	50				
	HW	tC/ha/yr	1.11	0.31	0.61	0.52	0.87	0.57			
		N	822	752	1013	1145	713	61			
Central States	SW	tC/ha/yr			0.47						
		N			52						
	HW	tC/ha/yr	1.13	0.97	0.38	0.19	0.08	-0.49			
		N	137	464	1006	1010	545	47			
Great Plains	SW	tC/ha/yr				-0.35	-0.64				
		N				51	55				
	HW	tC/ha/yr		0.68	0.41	0.04	-0.03				
		N		70	88	53	32				

Region	Group		0-20	21-40	41-60	61-80	81-120	121+	121-160	161-300	301+
Rocky Mountain- North	SW	tC/ha/yr	0.58	0.92	0.86	0.60	-0.26		-0.63	-1.00	
		N	385	144	161	396	900		451	400	
	WL	tC/ha/yr					0.05				
		N					35				
Rocky Mountain-South	SW	tC/ha/yr	0.20	0.16	0.37	0.21	-0.33		-0.84	-1.43	
		N	71	31	54	222	788		404	293	
	HW	tC/ha/yr	0.01	0.07	-0.08	-0.44	-0.88		-1.81		
		N	61	36	51	111	222		36		
	WL	tC/ha/yr	0.01	-0.02	0.08	0.06	0.01		-0.06	-0.03	-0.05
		N	454	162	234	443	1284		1138	1585	105
Pacific Northwest - East	SW	tC/ha/yr	0.73	1.09	0.76	0.46	0.27		0.36	0.28	0.43
		N	246	234	441	865	1145		408	405	42
Pacific Northwest - West	SW	tC/ha/yr	3.33	3.57	0.56	1.06	1.73		1.56	0.64	0.55
		N	420	562	282	273	408		276	539	202
	HW	tC/ha/yr	1.88	2.34	-0.55	0.88	0.62				
		N	96	64	73	66	41				
Pacific Southwest	SW	tC/ha/yr	1.92	2.99	1.25	1.09	0.58		0.30	-0.10	-0.77
		N	59	92	135	273	451		195	306	66
	HW	tC/ha/yr	0.31	1.88	1.13	0.67	0.22		-0.50	-0.57	
		N	34	68	129	141	160		73	40	
	WL	tC/ha/yr					-0.04				
		N					39				
