# Supplementary material for: Aboveground live tree carbon stock and change in forests of conterminous United States: influence of stand age
Source: Carbon Balance Manag. 2023 Apr 16;18:7. doi: 10.1186/s13021-023-00227-z (PMC10108445; doi:10.1186/s13021-023-00227-z)
Supplement: Supplementary file 3 — Additional file 3: Table S3. Forested area by region, type (softwood, hardwood, woodland), and age class (kha, thousand hectares). % = percentage of total forestland in that age class. Values less than one percent are displayed as zeroes; empty cells indicate no data for that category. [file 13021_2023_227_MOESM3_ESM.pdf]

Table S3. Forested area by region, type (softwood, hardwood, woodland), and age class (kha, thousand hectares). % = percentage of total forestland in that age class. Values less than one percent are displayed as zeroes; empty cells indicate no data for that category.

Region	Group		0-20	21-40	41-60	61-80	81-120	121+	121-160	161-300	301+
Northeast	SW	kha	252	1041	1017	1473	1543	243			
		%	1	3	3	4	5	1			
	HW	kha	909	2471	5420	8973	9921	540			
		%	3	7	16	26	29	2			
Northern Lake States	SW	kha	486	878	1221	1295	1271	335			
		%	2	4	6	6	6	2			
	HW	kha	2005	2341	3355	4682	3896	199			
		%	9	11	15	21	18	1			
South Central	SW	kha	8141	6884	2509	1408	343				
		%	15	12	4	3	1				
	HW	kha	6236	6151	9550	10582	3377	47			
		%	11	11	17	19	6	0			
	WL	kha	8	5	1	4	3				
		%	0	0	0	0	0				
Southeast	SW	kha	5172	5574	1785	1029	394	14			
		%	14	15	5	3	1	0			
	HW	kha	4378	3751	4126	4902	4131	281			
		%	12	10	11	14	11	1			
Central States	SW	kha	46	86	144	116	39	1			
		%	0	1	1	1	0	0			
	HW	kha	557	1387	3737	4653	3312	257			
		%	4	10	26	32	23	2			

Region	Group		0-20	21-40	41-60	61-80	81-120	121+	121-160	161-300	301+
Great Plains	SW	kha	136	334	234	199	289		54	9	
		%	0	1	0	0	1		0	0	
	HW	kha	1875	3861	6131	3864	1092		91	25	
		%	4	8	13	8	2		0	0	
	WL	kha	5122	9359	8197	3073	549		31		6
		%	11	19	17	6	1		0		0
Rocky Mountain-North	SW	kha	2464	1077	1040	1763	5023		2640	1907	139
		%	13	6	5	9	26		14	10	1
	HW	kha	296	76	59	91	131		12	6	
		%	2	0	0	0	1		0	0	
	WL	kha	56	78	120	163	232		110	75	2
		%	0	0	1	1	1		1	0	0
Rocky Mountain-South	SW	kha	906	504	282	877	4432		2485	1799	44
		%	2	1	1	2	10		6	4	0
	HW	kha	715	183	175	505	1056		217	17	
		%	2	0	0	1	2		1	0	
	WL	kha	2604	1172	1168	2155	5781		5412	7323	499
		%	6	3	3	5	14		13	17	1
Pacific Northwest - East	SW	kha	491	794	954	1674	3113		989	801	120
		%	5	8	10	17	32		10	8	1
	HW	kha	39	20	21	69	103		15	3	26
		%	0	0	0	1	1		0	0	0
	WL	kha	1	1	3		6		0		
		%	0	0	0		0		0		
Pacific Northwest - West	SW	kha	1410	2137	1266	842	1062		661	1074	560
		%	13	19	11	8	10		6	10	5
	HW	kha	368	391	394	272	226		42	25	49
		%	3	4	4	2	2		0	0	0

Region	Group		0-20	21-40	41-60	61-80	81-120	121+	121-160	161-300	301+
Pacific Southwest	SW	kha	267	402	643	1079	2233		954	1172	301
		%	2	3	5	8	17		7	9	2
	HW	kha	266	283	573	514	755		269	140	1842
		%	2	2	4	4	6		2	1	14
	WL	kha	11	4	1	31	55		50	85	440
		%	0	0	0	0	0		0	1	3
