# Supplementary material for: Aboveground live tree carbon stock and change in forests of conterminous United States: influence of stand age
Source: Carbon Balance Manag. 2023 Apr 16;18:7. doi: 10.1186/s13021-023-00227-z (PMC10108445; doi:10.1186/s13021-023-00227-z)
Supplement: Supplementary file 4 — Additional file 4: Table S4. Mean carbon density (metric tons C/hectare, tC/ha) by state, type (softwood, hardwood, woodland) and age class. Estimates are for aboveground live tree carbon. SEM = standard error of the mean. For states that span more than one region, carbon density is given for the entire state as well as the portion in each region. Values less than one percent are displayed as zeroes; empty cells indicate no data for that category. [file 13021_2023_227_MOESM4_ESM.pdf]

Table S4. Mean carbon density (metric tons C/hectare, tC/ha) by state, type (softwood, hardwood, woodland) and age class. Estimates are for aboveground live tree carbon. SEM = standard error of the mean. For states that span more than one region, carbon density is given for the entire state as well as the portion in each region. Values less than 0.1 are displayed as zeroes; empty cells indicate no data for that category.

State	Type		0-20	21-40	41-60	61-80	81-120	121+	121-160	161-300	300+
Alabama	All	tC/ha	25.8	60.1	70.5	82.5	87.8	69.2			
		SEM	0.5	0.8	1.2	1.4	2.8				
	SW	tC/ha	31.1	64.6	75.7	84.6	71.6				
		SEM	0.7	1.0	2.5	3.4	6.1				
	HW	tC/ha	18.3	53.4	68.7	82.1	90.3	69.2			
		SEM	0.7	1.1	1.3	1.5	3.0				
Arizona	All	tC/ha	1.9	3.5	5.5	13.7	23.3		18.5	17.3	20.5
		SEM	0.2	0.6	0.8	1	0.8		0.9	0.6	1.9
	SW	tC/ha	4.4	14.8	20.4	34.2	48.9		56.8	61.0	
		SEM	1.1	2.3	5.2	2.1	1.5		4.2	6.2	
	HW	tC/ha	7.5	4.9	49.9	33.3	83.9		63.9	3.3	
		SEM	2.4	1.4	17.2	9.2	16.5		10.9	0.8	
	WL	tC/ha	2.3	2.7	3.9	6.6	9.9		12.8	15.4	20.5
		SEM	0.3	0.5	0.4	0.5	0.4		0.5	0.4	1.9
Arkansas	All	tC/ha	21.9	52.7	58.8	66.9	76.7	100			
		SEM	0.8	1.1	1	1.1	2	3.7			
	SW	tC/ha	27.9	64.2	72.0	73.3	84.5				
		SEM	1.1	1.3	2.3	2.7	5.0				
	HW	tC/ha	13.8	37.3	54.3	66.1	75.4	100.0			
		SEM	0.9	1.5	1.1	1.2	2.2	3.7			
	WL	tC/ha			16.2	34.4	49.4				
		SEM				5.6	1.4				
California	All	tC/ha	8.1	49.8	84.7	77.1	91.4		110.7	117.9	49.2
		SEM	0.8	2.8	3.1	2.5	2		3.8	3.9	2.9
	SW	tC/ha	14.4	50.5	83.9	78.0	94.4		120.1	127.5	157.0
		SEM	2.0	3.5	4.4	3.0	2.4		4.3	4.4	20.4
	HW	tC/ha	10.3	49.4	85.7	79.1	88.5		96.4	99.4	41.3
		SEM	1.6	4.7	4.3	4.6	4.3		8.7	8.8	1.4
	WL	tC/ha	0.3	2.0	1.5	12.7	11.2		7.7	16.8	8.6
		SEM	0.2	0.6		3.3	1.9		1.6	1.9	0.7

State	Type		0-20	21-40	41-60	61-80	81-120	121+	121-160	161-300	300+
Colorado	All	tC/ha	6.5	10.2	16.1	25.1	37.2		42	36.9	31.2
		SEM	0.3	0.6	1.2	1.1	0.8		1.2	1.1	4.6
	SW	tC/ha	10.3	11.1	21.3	31.8	43.7		52.5	60.6	57.9
		SEM	0.8	1.1	1.8	1.6	1.0		1.6	2.0	16.3
	HW	tC/ha	9.6	16.7	30.1	34.2	46.2		63.2	54.5	
		SEM	0.9	2.5	3.4	2.2	1.9		4.6	22.8	
	WL	tC/ha	6.4	8.2	7.1	10.2	12.4		17.0	20.5	24.8
		SEM	0.4	0.7	0.5	0.6	0.5		0.7	0.5	2.8
Connecticut	All	tC/ha	25.6	28.9	63	90.7	98	101.6			
		SEM	10.4	4.5	5	3.3	2.8	8.3			
	SW	tC/ha	10.3	29.2	15.7	132.4	83.8				
		SEM				27.1	5.2				
	HW	tC/ha	41.5	28.9	64.1	89.0	98.5	101.6			
		SEM	15.8	4.5	5.0	3.3	2.9	8.3			
Delaware	All	tC/ha	29.9	58	81.4	86.6	104.8	82.6			
		SEM	11.3	5.2	7.4	5.3	6.5	24.8			
	SW	tC/ha	26.7	59.0	98.8	92.9	99.3				
		SEM	7.5	5.6	12.5	9.3	12.2				
	HW	tC/ha	35.8	57.2	74.7	85.5	105.2	82.6			
		SEM	21.0	8.1	8.4	6.0	6.9	24.8			
Florida	All	tC/ha	17	39	47.4	65.5	84.8	95.5			
		SEM	0.6	0.9	1.4	1.8	2.8	6.5			
	SW	tC/ha	23.7	45.7	48.3	54.0	65.1	26.3			
		SEM	0.9	1.2	2.1	2.8	5.1	4.4			
	HW	tC/ha	11.5	29.4	46.9	69.8	89.1	99.8			
		SEM	0.9	1.3	1.8	2.2	3.1	6.1			
Georgia	All	tC/ha	21.1	57.4	69	85.9	99.9	103.8			
		SEM	0.6	0.7	1.3	1.4	2.3	13.9			
	SW	tC/ha	27.2	62.1	73.6	87.9	93.8	39.6			
		SEM	0.8	0.9	2.4	3.4	7.8	11.5			
	HW	tC/ha	15.2	49.0	66.9	85.3	100.5	118.0			
		SEM	0.7	1.2	1.5	1.5	2.4	12.4			

State	Type		0-20	21-40	41-60	61-80	81-120	121+	121-160	161-300	300+
Idaho	All	tC/ha	5.9	18	38	58.5	69.3		65.4	64.6	45.1
		SEM	0.3	0.9	1.6	2.1	1.5		2.2	2.9	5.3
	SW	tC/ha	8.7	18.5	40.3	61.8	71.7		67.8	67.8	45.1
		SEM	0.4	1.0	1.7	2.2	1.6		2.2	3.0	5.3
	HW	tC/ha	7.8	16.6	24.9	30.7	43.5		29.8		
		SEM	1.0	3.4	3.5	3.9	5.4				
	WL	tC/ha	7.2	10.6	10.8	10.5	14.4		9.8	13.9	
		SEM	2.9	1.9	1.9	1.3	2.5		1.6	2.4	
Illinois	All	tC/ha	15.7	39.3	56.3	66.7	71.9	81.5			
		SEM	2.4	2.5	1.8	1.7	1.8	8.2			
	SW	tC/ha	9.1	79.7	77.2	85.9	60.6				
		SEM	2.1	20.6	9.0	11.2					
	HW	tC/ha	18.0	38.3	55.9	66.3	71.9	81.5			
		SEM	2.8	2.4	1.8	1.7	1.8	8.2			
Indiana	All	tC/ha	14.8	47.8	61	71.1	82.9	90.3			
		SEM	2.2	2.6	1.8	1.9	2.2	8.3			
	SW	tC/ha	6.8	18.1	68.4	55.1	40.4				
		SEM	2.1	2.1	6.3	5.5	9.5				
	HW	tC/ha	16.2	49.2	60.7	71.5	83.3	90.3			
		SEM	2.5	2.7	1.8	2.0	2.2	8.3			
Iowa	All	tC/ha	8	27.3	43.7	60.8	67.8	60.4			
		SEM	1.5	2	1.8	2.8	3.2	5.6			
	SW	tC/ha	14.3	12.9	25.7	57.4					
		SEM		2.5	3.0						
	HW	tC/ha	10.7	28.1	44.0	60.9	67.8	60.4			
		SEM	2.0	2.0	1.8	2.8	3.2	5.6			
Kansas	All	tC/ha	12.1	29.8	49	59.3	54.5				
		SEM	3.1	2.1	1.9	3.4	4.8				
	SW	tC/ha	2.4	9.2	26.7	15.5	22.6				
		SEM	1.4	2.6	3.9	3.7	4.7				
	HW	tC/ha	15.7	31.5	49.9	60.5	56.0				
		SEM	4.0	2.2	2.0	3.4	5.0				

State	Type		0-20	21-40	41-60	61-80	81-120	121+	121-160	161-300	300+
Kentucky	All	tC/ha	14.2	36.6	60.6	79.1	90.6	306.2			
		SEM	1.0	1.2	0.8	1.1	2.3				
	SW	tC/ha	14.8	33.5	59.4	68.4	54.4				
		SEM	3.4	3.5	5.7	11.5	7.3				
	HW	tC/ha	14.8	37.0	60.7	79.2	90.9	306.2			
		SEM	1.1	1.3	0.9	1.1	2.3				
Louisiana	All	tC/ha	22.2	57.4	66.7	77.4	92.7	85.4			
		SEM	0.7	1.1	1.6	1.8	4.4				
	SW	tC/ha	28.8	64.6	83.0	90.8	90.3				
		SEM	0.9	1.4	3.7	4.6	13.7				
	HW	tC/ha	14.7	47.7	62.5	75.1	92.9	85.4			
		SEM	0.8	1.5	1.7	2.0	4.6				
Maine	All	tC/ha	11.3	30.9	42.8	56.3	64.3	68.7			
		SEM	1.2	0.6	0.8	0.9	1.0	3.1			
	SW	tC/ha	15.1	33.0	43.5	51.1	56.4	63.6			
		SEM	1.8	0.8	1.4	1.6	1.5	3.8			
	HW	tC/ha	9.2	28.9	42.5	59.2	70.8	82.2			
		SEM	1.6	1.0	1.0	1.1	1.3	4.0			
Maryland	All	tC/ha	20.7	65	92.5	93.9	106.2	102.1			
		SEM	3.4	4.4	5.0	3.7	3.6	12.3			
	SW	tC/ha	24.3	74.4	88.9	95.9	85.5	81.2			
		SEM	4.1	5.8	5.6	17.8	20.5	5.7			
	HW	tC/ha	18.6	57.0	93.5	93.7	107.1	104.0			
		SEM	6.4	6.2	6.3	3.7	3.6	13.3			
Massachusetts	All	tC/ha	13.1	29.6	59	85.8	98.1	118.9			
		SEM	7.5	6.7	4.6	2.3	2.0	9.3			
	SW	tC/ha		22.4	47.3	90.7	107.3	112.3			
		SEM		5.5	11.3	7.8	5.3	7.4			
	HW	tC/ha	19.5	32.1	61.2	85.0	95.9	127.1			
		SEM	11.6	8.7	5.0	2.4	2.1	16.8			
Michigan	All	tC/ha	10.8	29	46.1	57.8	66.6	75.5			
		SEM	0.7	1.0	1.0	0.8	1.0	4.3			
	SW	tC/ha	11.2	26.2	42.2	48.3	53.0	67.7			
		SEM	1.4	1.6	1.7	1.6	1.6	5.5			
	HW	tC/ha	11.9	30.2	47.5	60.4	70.8	83.7			
		SEM	0.8	1.2	1.2	0.9	1.1	6.3			

State	Type		0-20	21-40	41-60	61-80	81-120	121+	121-160	161-300	300+
Minnesota	All	tC/ha	11.5	28.4	36.2	42.1	47.2	41.5			
		SEM	0.4	0.5	0.7	0.7	0.8	1.8			
	SW	tC/ha	6.6	26.5	27.9	29.0	33.4	35.1			
		SEM	0.8	1.1	1.1	1.1	1.2	1.7			
	HW	tC/ha	13.3	29.1	40.2	47.6	55.9	59.7			
		SEM	0.4	0.5	0.8	0.8	1.0	3.8			
Mississippi	All	tC/ha	27.4	68.0	74.9	89.0	90.8				
		SEM	0.8	0.9	1.3	1.7	4.2				
	SW	tC/ha	34.7	77.2	85.1	94.4	106.0				
		SEM	1.1	1.1	2.6	3.3	9.0				
	HW	tC/ha	19.8	52.8	71.1	87.8	87.3				
		SEM	1.0	1.1	1.5	2.0	4.6				
Missouri	All	tC/ha	14.5	32.6	47.0	51.6	58.4	62.2			
		SEM	1.7	1.1	0.8	0.6	0.8	3.6			
	SW	tC/ha	9.9	27.6	40.1	47.4	59.5	86.6			
		SEM	4.4	4.0	3.1	3.5	6.8				
	HW	tC/ha	17.3	33.1	47.4	51.7	58.4	62.0			
		SEM	2.1	1.1	0.8	0.6	0.8	3.6			
Montana	All	tC/ha	5.1	13.6	23.0	32.5	46.3		53.8	60.3	51.4
		SEM	0.2	0.6	1.1	1.4	0.9		1.3	1.5	6.3
	SW	tC/ha	7.7	14.4	26.3	37.4	48.7		55.7	61.6	53.4
		SEM	0.3	0.7	1.2	1.5	1.0		1.3	1.5	6.2
	HW	tC/ha	4.7	14.9	23.9	23.5	34.8		28.4	64.6	
		SEM	0.9	3.1	4.3	3.4	5.1		7.6	26.5	
	WL	tC/ha	2.9	3.5	4.8	6.5	10.4		11.9	11.4	0
		SEM	1.0	0.5	0.7	0.7	0.8		1.5	1.8	
Nebraska	All	tC/ha	4.6	25.2	31.3	54.1	48.9		129.6		
		SEM	1.6	2.9	2.9	4.8	5.1				
	SW	tC/ha	9.2	16.7	16.9	22.8	31.4				
		SEM	3.2	4.3	3.0	3.0	4.3				
	HW	tC/ha	8.4	28.7	39.1	64.2	60.8		129.6		
		SEM	4.3	3.5	3.8	5.8	7.7				
	WL	tC/ha			23.6		26.0				
		SEM									

State	Type		0-20	21-40	41-60	61-80	81-120	121+	121-160	161-300	300+
Nevada	All	tC/ha	0.8	4.3	7.2	8.9	11.1		13.8	15.8	21.3
		SEM	0.1	0.6	1.0	0.7	0.4		0.5	0.5	3.9
	SW	tC/ha	4.3	1.2	29.6	21.7	38.0		33.1	41.7	78.8
		SEM	1.6		10.9	9.4	5.5		5.1	6.2	15.9
	HW	tC/ha	4.0	9.0	17.7	28.1	23.1		22.7		
		SEM	1.0	1.6	3.1	6.2	6.6		5.7		
	WL	tC/ha	1.1	3.1	5.3	7.6	9.9		13.0	14.4	13.0
		SEM	0.2	0.4	0.5	0.4	0.3		0.4	0.4	1.3
New Hampshire	All	tC/ha	15.1	33.1	60.2	76.0	89.0	106.9			
		SEM	2.4	3.0	2.3	1.6	2.0	12.0			
	SW	tC/ha	21.8	25.7	56.7	73.9	83.7	53.1			
		SEM	6.5	3.7	5.1	3.6	3.8	3.2			
	HW	tC/ha	14.9	36.0	60.8	76.7	90.8	125.3			
		SEM	2.6	3.9	2.5	1.8	2.3	6.4			
New Jersey	All	tC/ha	7.6	41.5	60.1	73.8	81.8	91.4			
		SEM	1.5	3.6	2.8	2.6	2.5	6.8			
	SW	tC/ha	7.9	39.9	43.6	50.9	47.8	38.4			
		SEM	2.6	6.6	2.7	2.4	2.8	4.1			
	HW	tC/ha	7.6	42.5	67.8	82.1	89.3	99.9			
		SEM	2.2	4.1	3.7	3.1	2.7	6.7			
New Mexico	All	tC/ha	1.8	2.6	5.6	13.6	21.3		19.6	18.4	14.5
		SEM	0.2	0.3	0.4	0.8	0.6		0.8	0.9	1.7
	SW	tC/ha	13.0	7.3	14.1	30.1	40.4		47.2	55.6	
		SEM	2.8	1.4	1.9	2.2	1.1		2.1	4.3	
	HW	tC/ha	5.5	6.3	15.8	43.1	50.8		46.0	64.1	
		SEM	1.8	3.0	6.4	5.7	4.1		9.0		
	WL	tC/ha	2.2	2.4	4.8	7.3	10.2		11.5	12.8	14.5
		SEM	0.2	0.3	0.4	0.4	0.4		0.4	0.4	1.7
New York	All	tC/ha	11.5	32.9	61.3	80.2	89.5	93.5			
		SEM	1.9	1.6	1.2	1	1.1	3.4			
	SW	tC/ha	13.5	32.4	58.5	73.9	77.9	82.6			
		SEM	8.9	4.2	3.5	3.0	3.2	6.1			
	HW	tC/ha	16.0	33.0	61.7	81.1	90.9	96.9			
		SEM	2.9	1.7	1.3	1.1	1.1	3.9			

State	Type		0-20	21-40	41-60	61-80	81-120	121+	121-160	161-300	300+
North Carolina	All	tC/ha	23.6	64	76.7	95.3	105.9	113.7			
		SEM	0.8	1.1	1.5	1.5	2	7			
	SW	tC/ha	31.0	72.1	77.7	92.5	88.9	101.5			
		SEM	1.2	1.6	2.8	5.3	6.5	21.2			
	HW	tC/ha	17.7	52.9	76.3	95.7	107.2	115.2			
		SEM	0.9	1.4	1.7	1.5	2.1	7.4			
North Dakota	All	tC/ha	6.3	21.9	27.3	27.0	38.3		103.7		
		SEM	2.1	4.9	3.1	2.9	4.4				
	SW	tC/ha					6.1				
		SEM									
	HW	tC/ha	9.0	22.3	29.1	31.3	45.1		103.7		
		SEM	2.8	5.0	3.3	3.2	4.9				
	WL	tC/ha		0.8	9.5	11.4	13.3				
		SEM		0.2	4.3	2.0	3.0				
Ohio	All	tC/ha	14.3	40.9	64.8	82.3	95.5	121.5			
		SEM	1.5	1.9	1.5	1.6	2.5	12.4			
	SW	tC/ha	28.3	56.1	55.8	88.1	120.8				
		SEM	6.9	9.7	8.2	8.5	8.5				
	HW	tC/ha	14.4	40.6	65.1	82.1	95.4	121.5			
		SEM	1.6	1.9	1.5	1.7	2.5	12.4			
Oklahoma	All	tC/ha	7.2	22.6	32.8	42.3	47.1				
		SEM	0.3	0.6	0.6	0.7	1.8				
	SW	tC/ha	14.8	32.6	38.1	52.9	73.6				
		SEM	1.0	1.7	1.9	3.0	6.6				
	HW	tC/ha	6.8	21.1	33.4	41.7	44.6				
		SEM	0.4	0.5	0.6	0.7	1.8				
	WL	tC/ha	0.8	5.4	9.8	5.9					
		SEM	0.2	0.6	2.2	1.3					
Oklahoma – Great Plains	All	tC/ha	3.9	16.7	27.8	38.2	36.8				
		SEM	0.3	0.5	0.7	1.1	2.1				
	SW	tC/ha	4.6	11.5	18.9	20.1					
		SEM	1.1	1.2	1.8	4.1					
	HW	tC/ha	5.6	19.4	30.0	38.9	36.8				
		SEM	0.5	0.6	0.7	1.1	2.1				
	WL	tC/ha	0.8	5.4	9.8	5.9					
		SEM	0.2	0.6	2.2	1.3					

State	Type		0-20	21-40	41-60	61-80	81-120	121+	121-160	161-300	300+
Oklahoma – South Central	All	tC/ha	11.8	30.6	39.3	45.5	52.1				
		SEM	0.6	1.0	1.0	0.9	2.3				
	SW	tC/ha	17.3	43.5	47.8	57.8	73.6				
		SEM	1.1	2.0	1.9	3.0	6.6				
	HW	tC/ha	8.8	23.8	37.7	44.1	48.9				
		SEM	0.7	0.9	1.1	1.0	2.3				
Oregon	All	tC/ha	12.3	66.1	95.7	72.4	84.3		134.3	163.5	195.8
		SEM	0.7	1.7	2.6	2.6	2		4.5	4.4	9.2
	SW	tC/ha	16.7	65.9	98.3	69.3	83.7		135.4	163.3	231.0
		SEM	1.1	1.8	3.0	2.8	2.1		4.6	4.5	9.3
	HW	tC/ha	12.5	67.6	82.7	100.0	94.8		108.9	172.1	48.9
		SEM	2.0	4.9	4.7	7.1	6.5		22.3	20.0	8.1
	WL	tC/ha		0.5	8.7		4.8		13.0		
		SEM		0.2	5.6		3.1				
Oregon – Pacific Northwest- East	All	tC/ha	6.1	18.7	25.8	32.3	50.2		60.8	74.8	73.6
		SEM	0.5	0.7	1.1	1.0	1.2		2.6	3.6	11.0
	SW	tC/ha	11.3	18.9	26.1	32.5	50.7		61.2	74.8	89.9
		SEM	0.9	0.7	1.1	1.0	1.2		2.6	3.6	13.2
	HW	tC/ha	2.8	8.4	8.8	21.4	33.7		30.4	22.0	29.1
		SEM	1.5	2.4	3.1	7.8	5.3		16.1		9.3
	WL	tC/ha		0.5	8.7		4.8		13.0		
		SEM		0.2	5.6		3.1				
Oregon - Pacific Northwest- West	All	tC/ha	15.6	85.2	135.1	144.3	169.5		224.7	235.0	227.9
		SEM	1.1	2.0	3.1	5.1	4.8		6.9	5.3	9.8
	SW	tC/ha	18.4	88.0	150.3	156.8	185.8		233.6	237.7	263.7
		SEM	1.4	2.1	3.5	6.1	5.5		6.9	5.4	8.7
	HW	tC/ha	13.3	69.7	84.9	107.5	110.2		122.0	172.2	57.0
		SEM	2.2	5.0	4.7	7.3	7.2		24.3	20.0	10.2
Pennsylvania	All	tC/ha	18.5	38.8	62.4	78.9	91.2	97.4			
		SEM	1.6	1.6	1.4	1.2	1.0	5.9			
	SW	tC/ha	29.8	39.4	61.5	66.9	73.7	65.6			
		SEM	9.9	6.2	5.3	6.3	5.0	12.1			
	HW	tC/ha	20.6	38.8	62.4	79.3	91.7	98.7			
		SEM	1.8	1.7	1.4	1.2	1.0	6.1			

State	Type		0-20	21-40	41-60	61-80	81-120	121+	121-160	161-300	300+
Rhode Island	All	tC/ha	3.1	41.5	60.0	82.3	93.4				
		SEM	2.7	11.3	6.6	3.3	5.3				
	SW	tC/ha		14.1	42.6	94.0	96.7				
		SEM			4.6	5.6	13.0				
	HW	tC/ha	12.6	52.4	61.7	80.6	93.0				
		SEM		8.3	7.1	3.6	5.7				
South Carolina	All	tC/ha	23.3	61.8	77.8	94.8	98.4	119.2			
		SEM	0.8	1.0	1.8	2.3	3.7	39.7			
	SW	tC/ha	27.4	64.5	79.7	91.6	84.5				
		SEM	1.2	1.2	3.1	5.8	6.6				
	HW	tC/ha	20.3	56.0	76.6	95.5	101.8	119.2			
		SEM	1.1	1.6	2.2	2.4	4.3	39.7			
South Dakota	All	tC/ha	3.9	15.8	25.2	31.6	32.8		32.1	42.6	
		SEM	0.7	1.7	2.3	2.9	1.5		3.0	7.5	
	SW	tC/ha	8.0	15.3	24.7	30.6	32.7		32.2	42.6	
		SEM	2.0	2.1	3.2	4.1	1.5		3.1	7.5	
	HW	tC/ha	5.7	17.8	26.9	37.0	35.2		31.9		
		SEM	2.7	2.6	3.7	4.0	4.6		11.7		
	WL	tC/ha	2.9	12.1	17.2	8.3	18.3		28.8		
		SEM		7.1	2.7	1.1	3.0				
Tennessee	All	tC/ha	22.5	51.6	66.4	81.7	91.2	91.1			
		SEM	1.1	1.6	1.3	1.1	1.6	9.5			
	SW	tC/ha	32.6	57.6	64.4	79.4	89.6				
		SEM	2.4	3.6	5.0	5.3	8.9				
	HW	tC/ha	18.6	49.4	66.5	81.8	91.2	91.1			
		SEM	1.0	1.7	1.4	1.1	1.6	9.5			
Texas	All	tC/ha	6.6	14.8	18.0	25.7	26.7		20.0	36.1	3.4
		SEM	0.2	0.2	0.3	0.5	1.2		3.1	11.5	
	SW	tC/ha	23.7	54.4	75.0	79.9	77.6				
		SEM	0.6	0.9	2.0	3.1	9.6				
	HW	tC/ha	11.8	22.8	26.3	32.8	30.2		21.4	36.1	
		SEM	0.4	0.5	0.5	0.8	1.5		3.5	11.5	
	WL	tC/ha	1.4	4.5	7.0	11.2	13.5		16.1		3.4
		SEM	0.1	0.1	0.1	0.3	0.8		6.6		

State	Type		0-20	21-40	41-60	61-80	81-120	121+	121-160	161-300	300+
Texas – Great Plains	All	tC/ha	1.7	6.7	11.1	16.8	20.8		20.0	36.1	3.4
		SEM	0.1	0.1	0.2	0.3	0.9		3.1	11.5	
	SW	tC/ha	18.8	23.9	22.9	19.5	15.9				
		SEM	4.0	3.4	3.6	2.9	2.3				
	HW	tC/ha	6.6	14.7	18.8	23.3	26.4		21.4	36.1	
		SEM	0.4	0.5	0.4	0.6	1.3		3.5	11.5	
	WL	tC/ha	1.4	4.5	7.0	11.2	13.5		16.1		3.4
		SEM	0.1	0.1	0.1	0.3	0.8		6.6		
Texas – South Central	All	tC/ha	19.4	47.3	57.5	70.2	92.1				
		SEM	0.4	0.7	1.0	1.6	5.9				
	SW	tC/ha	23.8	55.8	77.0	90.9	111.0				
		SEM	0.6	0.9	2.0	2.9	9.2				
	HW	tC/ha	15.5	37.5	48.4	62.4	74.9				
		SEM	0.6	0.9	1.0	1.8	6.2				
	WL	tC/ha	3.1	6.9							
		SEM	0.2								
Utah	All	tC/ha	4.2	11.7	13.7	16.6	25.2		21.1	19.8	21.3
		SEM	0.3	0.8	1.3	1.0	0.9		0.9	0.5	2.3
	SW	tC/ha	8.6	13.7	18.0	29.8	43.8		46.1	43.4	
		SEM	1.2	1.5	2.5	2.7	2.2		3.0	2.7	
	HW	tC/ha	5.0	13.4	23.9	23.4	45.4		40.2	74.9	
		SEM	0.6	1.6	3.2	2.1	2.8		5.1	4.3	
	WL	tC/ha	5.1	9.6	7.6	11.0	13.7		13.6	16.9	21.3
		SEM	0.4	1.1	0.8	0.9	0.5		0.5	0.4	2.3
Vermont	All	tC/ha	18.1	35.3	57.8	77.5	92.2	100.7			
		SEM	3.1	3.0	2.0	1.6	1.7	9.4			
	SW	tC/ha	17.2	33.5	55.3	80.3	91.0	93.8			
		SEM	5.9	6.3	5.3	5.0	3.5	11.5			
	HW	tC/ha	19.7	35.6	58.3	76.9	92.3	114.2			
		SEM	3.7	3.3	2.2	1.7	1.8	8.9			
Virginia	All	tC/ha	28.1	69.8	81.0	88.4	95.5	94.4			
		SEM	1.0	1.4	1.6	1.4	1.3	3.6			
	SW	tC/ha	37.6	84.7	96.3	105.3	88.7				
		SEM	1.7	2.4	4.7	6.5	7.3				
	HW	tC/ha	21.7	59.2	78.0	87.1	95.8	94.4			
		SEM	1.2	1.6	1.7	1.5	1.3	3.6			

State	Type		0-20	21-40	41-60	61-80	81-120	121+	121-160	161-300	300+
Washington	All	tC/ha	13.8	72.2	98.9	105.6	97.1		118.3	181.6	236.7
		SEM	0.8	1.7	3.3	3.4	2.8		4.6	5.2	9.5
	SW	tC/ha	17.1	74.0	101.4	104.7	98.2		119.6	182.3	241.8
		SEM	1.2	1.9	3.8	3.8	3.0		4.7	5.2	9.4
	HW	tC/ha	20.4	60.9	86.4	111.4	81.7		56.5	41.2	36.7
		SEM	2.3	2.9	5.5	7.9	9.4		19.5	22.0	7.8
Washington - Pacific Northwest - East	All	tC/ha	4.7	24.3	42.5	55.7	61.2		80.1	101.7	108.9
		SEM	0.4	1.2	2.5	2.0	1.7		3.5	5.2	15.9
	SW	tC/ha	7.5	24.2	42.4	57.1	62.5		81.3	102.6	117.2
		SEM	0.9	1.3	2.5	2.0	1.8		3.5	5.2	17.2
	HW	tC/ha	3.4	25.7	45.9	39.2	35.4		25.0	11.8	32.9
		SEM	1.8	4.9	19.1	7.4	5.1		10.8	1.3	9.9
Washington - Pacific Northwest- West	All	tC/ha	19.4	83.6	135.0	171.4	187.5		188.0	233.3	265.3
		SEM	1.2	1.9	4.1	5.5	6.7		10.1	6.7	9.5
	SW	tC/ha	20.8	87.3	149.7	180.2	195.2		189.4	233.6	267.3
		SEM	1.5	2.1	4.8	6.7	7.3		10.2	6.7	9.5
	HW	tC/ha	22.6	63.3	90.2	142.9	129.3		118.4	113.7	46.0
		SEM	2.5	3.0	5.6	7.8	12.9		4.7	10.5	6.8
West Virginia	All	tC/ha	18.5	44.4	71.2	85.2	95.0	97.3			
		SEM	2.8	2.5	1.6	1.3	1.4	6.1			
	SW	tC/ha	18.9	51.6	43.0	62.7	65.2	31.1			
		SEM	5.3	10.7	4.9	5.6	6.7	17.4			
	HW	tC/ha	19.5	44.1	72.2	85.7	95.4	99.6			
		SEM	3.2	2.6	1.6	1.3	1.4	5.6			
Wisconsin	All	tC/ha	12.1	30.1	42.4	54.1	64.7	78.6			
		SEM	0.5	0.6	0.6	0.6	0.8	4.0			
	SW	tC/ha	17.9	31.9	39.8	42.5	50.9	80.9			
		SEM	1.6	1.2	1.3	1.7	2.2	6.2			
	HW	tC/ha	12.0	29.5	43.2	56.2	66.9	76.5			
		SEM	0.5	0.7	0.7	0.7	0.8	5.1			

State	Type		0-20	21-40	41-60	61-80	81-120	121+	121-160	161-300	300+
Wyoming	All	tC/ha	5.6	11.3	17.7	25.6	37.3		45.5	48	30.9
		SEM	0.4	0.8	2.5	1.9	1.2		1.8	2.4	7.9
	SW	tC/ha	8.3	12.0	20.3	28.7	40.0		50.0	56.0	48.5
		SEM	0.7	0.9	3.2	2.2	1.2		1.8	2.6	6.5
	HW	tC/ha	7.0	11.4	20.1	30.0	36.9		51.2		
		SEM	0.8	2.0	5.6	5.0	4.4		14.5		
	WL	tC/ha	0.7	4.0	4.3	8.1	9.2		9.7	12.6	6.4
		SEM	0.3	1.1	0.9	1.5	1.9		1.3	1.6	0.7
