# Supplementary material for: Aboveground live tree carbon stock and change in forests of conterminous United States: influence of stand age
Source: Carbon Balance Manag. 2023 Apr 16;18:7. doi: 10.1186/s13021-023-00227-z (PMC10108445; doi:10.1186/s13021-023-00227-z)
Supplement: Supplementary file 5 — Additional file 5: Table S5. Carbon accumulation rates (metric tons C/hectare/year, tC/ha/yr) by state, type (softwood, hardwood, woodland) and age class. Estimates are for aboveground live tree carbon. N = number of paired plots on which the estimate is based; data are shown only if N ≥ 30. Categories are omitted if no bins meet the N≥ 30 cutoff; Delaware, Nebraska, North Dakota, Rhode Island, Wyoming, and the Great Plains portions of Oklahoma and Texas are not represented in this table because no categories met the sample size threshold. Note that the error of the estimate decreases with increasing N. For states that span more than one region, rates are given for the entire state as well as the portion in each region (if sufficient data are available). [file 13021_2023_227_MOESM5_ESM.pdf]

Table S5. Carbon accumulation rates (metric tons C/hectare/year, tC/ha/yr) by state, type (softwood, hardwood, woodland) and age class. Estimates are for aboveground live tree carbon. N = number of paired plots on which the estimate is based; data are shown only if N ≥ 30. Categories are omitted if no bins meet the N≥ 30 cutoff; Delaware, Nebraska, North Dakota, Rhode Island, Wyoming, and the Great Plains portions of Oklahoma and Texas are not represented in this table because no categories met the sample size threshold. Note that the error of the estimate decreases with increasing N. For states that span more than one region, rates are given for the entire state as well as the portion in each region (if sufficient data are available).

State	Type		0-20	21-40	41-60	61-80	81-120	121+	121-160	161-300	300+
Alabama	All	tC/ha/yr	2.08	0.28	0.04	-0.29	0.10				
		N	832	371	438	215	61				
	SW	tC/ha/yr	2.32	0.25	-0.57	-1.09					
		N	603	189	129	47					
	HW	tC/ha/yr	1.47	0.31	0.30	-0.06	0.46				
		N	229	182	309	168	53				
Arizona	All	tC/ha/yr	0.14	0.08	0.08	0.07	0.04		-0.07	-0.10	-0.06
		N	66	41	71	217	616		433	521	30
	SW	tC/ha/yr				0.17	0.20		-0.08		
		N				75	188		47		
	WL	tC/ha/yr	0.10	0.04	0.05	0.01	0.01		-0.06	-0.08	-0.06
		N	58	33	60	137	421		383	501	30
Arkansas	All	tC/ha/yr	2.75	0.47	0.34	0.11	0.30				
		N	346	317	558	522	79				
	SW	tC/ha/yr	3.20	0.44	-0.04	-0.22					
		N	249	161	130	69					
	HW	tC/ha/yr	1.57	0.51	0.46	0.16	0.11				
		N	97	156	428	453	63				
California	All	tC/ha/yr	1.32	2.50	1.17	0.90	0.45		0.07	-0.15	-0.64
		N	94	161	268	437	650		293	368	73
	SW	tC/ha/yr	1.92	2.99	1.25	1.09	0.58		0.30	-0.10	-0.77
		N	59	92	135	273	451		195	306	66
	HW	tC/ha/yr	0.31	1.88	1.13	0.67	0.22		-0.50	-0.57	
		N	34	68	129	141	160		73	40	
	WL	tC/ha/yr					-0.04				
		N					39				

State	Type		0-20	21-40	41-60	61-80	81-120	121+	121-160	161-300	300+
Colorado	All	tC/ha/yr	0.04	0.05	0.10	-0.06	-0.61		-0.77	-0.72	
		N	216	73	78	197	621		351	374	
	SW	tC/ha/yr	0.17			0.17	-0.67		-1.06	-1.64	
		N	38			86	353		214	162	
	HW	tC/ha/yr	-0.05			-0.47	-1.03				
		N	32			58	142				
	WL	tC/ha/yr	0.03	-0.02	0.11	0.03	0.04		-0.02	-0.02	
		N	146	42	30	53	126		118	212	
Connecticut	All	tC/ha/yr				0.77	0.98				
		N				58	64				
	HW	tC/ha/yr				0.77	0.98				
		N				58	64				
Florida	All	tC/ha/yr	1.24	0.26	0.41	0.63	1.00				
		N	384	268	229	165	95				
	SW	tC/ha/yr	1.58	0.21	0.28	0.22					
		N	269	141	75	41					
	HW	tC/ha/yr	0.44	0.32	0.47	0.76	0.94				
		N	115	127	154	124	85				
Georgia	All	tC/ha/yr	1.85	0.05	0.09	0.21	0.82				
		N	851	505	359	297	122				
	SW	tC/ha/yr	2.27	0.30	0.04	-0.04					
		N	592	283	118	51					
	HW	tC/ha/yr	0.90	-0.27	0.12	0.27	0.85				
		N	259	222	241	246	114				
Idaho	All	tC/ha/yr	0.58	1.27	1.01	0.77	-0.15		-0.48	-1.21	
		N	162	75	101	226	395		197	130	
	SW	tC/ha/yr	0.63	1.39	1.06	0.84	-0.15		-0.51	-1.21	
		N	147	66	91	202	373		190	125	
Illinois	All	tC/ha/yr		1.40	0.54	0.29	0.03				
		N		70	126	105	85				
	HW	tC/ha/yr		1.38	0.51	0.35	0.04				
		N		67	121	103	84				
Indiana	All	tC/ha/yr		0.99	0.46	0.24	0.50				
		N		72	143	130	84				
	HW	tC/ha/yr		1.00	0.49	0.24	0.50				
		N		66	138	127	84				

State	Type		0-20	21-40	41-60	61-80	81-120	121+	121-160	161-300	300+
Iowa	All	tC/ha/yr		0.66	0.35	0.72	0.46				
		N		39	64	51	30				
	HW	tC/ha/yr		0.64	0.35	0.72	0.46				
		N		38	63	51	30				
Kansas	All	tC/ha/yr		0.72	0.61						
		N		51	39						
	HW	tC/ha/yr		0.72	0.61						
		N		50	39						
Kentucky	All	tC/ha/yr	1.38	0.95	0.65	0.41	-0.32				
		N	40	214	450	247	74				
	HW	tC/ha/yr	1.21	0.97	0.64	0.40	-0.32				
		N	38	203	445	245	74				
Louisiana	All	tC/ha/yr	2.32	-0.05	-0.29						
		N	110	66	89						
	SW	tC/ha/yr	2.53	-0.36	-0.23						
		N	83	33	31						
	HW	tC/ha/yr		0.26	-0.32						
		N		33	58						
Maine	All	tC/ha/yr	1.61	1.17	0.38	-0.05	-0.19	-0.33			
		N	194	419	488	601	478	61			
	SW	tC/ha/yr	1.67	1.38	0.41	0.36	0.12	0.16			
		N	125	219	159	172	219	39			
	HW	tC/ha/yr	1.52	0.94	0.37	-0.21	-0.46				
		N	69	200	329	429	259				
Maryland	All	tC/ha/yr				0.81	-0.08				
		N				43	38				
	HW	tC/ha/yr				0.78	0.23				
		N				37	36				
Massachusetts	All	tC/ha/yr			1.09	0.68	0.95				
		N			35	122	72				
	HW	tC/ha/yr			1.15	0.64	0.89				
		N			30	107	58				

State	Type		0-20	21-40	41-60	61-80	81-120	121+	121-160	161-300	300+
Michigan	All	tC/ha/yr	1.00	0.70	0.35	0.22	0.21				
		N	165	280	589	713	358				
	SW	tC/ha/yr	1.02	0.72	0.58	0.40	0.47				
		N	53	94	165	160	108				
	HW	tC/ha/yr	1.00	0.70	0.27	0.17	0.10				
		N	112	186	424	553	250				
Minnesota	All	tC/ha/yr	1.08	0.89	0.07	-0.07	0.07	0.29			
		N	455	458	591	717	527	113			
	SW	tC/ha/yr	0.96	0.88	0.33	0.29	0.08	0.31			
		N	78	145	183	230	246	90			
	HW	tC/ha/yr	1.10	0.90	-0.04	-0.24	0.07				
		N	377	313	408	487	281				
Mississippi	All	tC/ha/yr	3.16	0.90	0.99	0.26					
		N	629	316	331	152					
	SW	tC/ha/yr	3.69	1.24	0.96	-0.36					
		N	446	162	91	36					
	HW	tC/ha/yr	1.86	0.54	1.00	0.45					
		N	183	154	240	116					
Missouri	All	tC/ha/yr	1.21	0.77	0.28	0.14	-0.07				
		N	72	196	490	555	263				
	SW	tC/ha/yr			0.24						
		N			34						
	HW	tC/ha/yr	1.19	0.76	0.28	0.13	-0.06				
		N	63	182	456	545	258				
Montana	All	tC/ha/yr	0.51	0.49	0.52	0.25	-0.32		-0.73	-0.90	
		N	259	88	91	218	561		273	281	
	SW	tC/ha/yr	0.55	0.53	0.61	0.36	-0.33		-0.72	-0.90	
		N	238	78	70	194	527		261	275	
Nevada	All	tC/ha/yr			0.19	0.06	0.07		0.02	-0.01	
		N			32	53	202		173	227	
	WL	tC/ha/yr				0.07	0.08		0.05	0.06	
		N				51	195		167	214	

State	Type		0-20	21-40	41-60	61-80	81-120	121+	121-160	161-300	300+
New Hampshire	All	tC/ha/yr			0.69	0.40	0.49				
		N			155	203	116				
	SW	tC/ha/yr				0.55					
		N				52					
	HW	tC/ha/yr			0.73	0.34	0.58				
		N			126	151	92				
New Jersey	All	tC/ha/yr			0.67	0.64	0.37				
		N			46	57	52				
	HW	tC/ha/yr			0.57	0.60	0.36				
		N			30	39	43				
New Mexico	All	tC/ha/yr	0.04	0.00	0.05	0.10	-0.22		-0.47	-0.60	
		N	103	48	55	121	342		212	180	
	SW	tC/ha/yr					-0.14		-0.67	-1.31	
		N					115		55	30	
	WL	tC/ha/yr	0.02	-0.01	0.03	0.06	-0.19		-0.40	-0.14	
		N	91	46	51	86	219		156	148	
New York	All	tC/ha/yr	0.97	1.11	0.73	0.38	0.32	0.64			
		N	64	194	451	655	431	36			
	SW	tC/ha/yr			0.68	0.30	0.20				
		N			71	69	42				
	HW	tC/ha/yr	0.88	1.10	0.74	0.39	0.33				
		N	58	174	380	586	389				
North Carolina	All	tC/ha/yr	2.52	0.16	0.68	0.55	0.58				
		N	407	298	295	311	162				
	SW	tC/ha/yr	3.10	-0.02	0.27	-0.43					
		N	260	169	62	39					
	HW	tC/ha/yr	1.48	0.39	0.79	0.70	0.72				
		N	147	129	233	272	147				
Ohio	All	tC/ha/yr	1.34	1.14	0.49	0.07	-0.01				
		N	39	113	235	184	89				
	HW	tC/ha/yr	1.23	1.15	0.46	0.07	-0.01				
		N	34	111	228	184	89				

State	Type		0-20	21-40	41-60	61-80	81-120	121+	121-160	161-300	300+
Oklahoma – South Central	All	tC/ha/yr	1.37	-0.07	0.08	0.07					
		N	73	78	97	93					
	SW	tC/ha/yr	1.94								
		N	40								
	HW	tC/ha/yr	0.68	-0.12	-0.05	-0.01					
		N	33	52	83	80					
Oregon	All	tC/ha/yr	2.03	2.67	0.56	0.62	0.75		1.01	0.68	1.03
		N	486	523	562	813	1128		492	644	123
	SW	tC/ha/yr	2.14	2.69	0.60	0.63	0.76		1.03	0.69	1.33
		N	417	475	510	763	1083		476	629	120
	HW	tC/ha/yr	1.31	2.47	0.22	0.49	0.61				
		N	69	48	52	50	45				
Oregon – Pacific Northwest- East	All	tC/ha/yr	0.71	0.90	0.70	0.48	0.40		0.53	0.51	
		N	187	162	325	623	811		282	289	
	SW	tC/ha/yr	0.72	0.91	0.70	0.50	0.40		0.54	0.51	
		N	184	159	323	614	802		279	289	
Oregon - Pacific Northwest- West	All	tC/ha/yr	2.85	3.47	0.38	1.08	1.67		1.67	0.81	1.01
		N	299	361	237	190	317		210	355	110
	SW	tC/ha/yr	3.27	3.59	0.42	1.18	1.80		1.73	0.84	1.33
		N	233	316	187	149	281		197	340	107
	HW	tC/ha/yr	1.36	2.61	0.23	0.70	0.68				
		N	66	45	50	41	36				
Pennsylvania	All	tC/ha/yr	1.36	1.18	0.93	0.46	0.25				
		N	77	130	266	515	528				
	HW	tC/ha/yr	1.33	1.18	0.94	0.48	0.28				
		N	73	121	255	500	515				
South Carolina	All	tC/ha/yr	2.06	-0.01	0.38	0.09	0.03				
		N	449	309	191	161	54				
	SW	tC/ha/yr	2.49	-0.08	-0.34	0.57					
		N	308	202	59	35					
	HW	tC/ha/yr	1.14	0.12	0.70	-0.04	0.78				
		N	141	107	132	126	44				
South Dakota	All	tC/ha/yr			0.16	-0.28	-0.68				
		N			32	49	64				
	SW	tC/ha/yr				-0.38	-0.72				
		N				40	50				

State	Type		0-20	21-40	41-60	61-80	81-120	121+	121-160	161-300	300+
Tennessee	All	tC/ha/yr	2.22	1.10	0.69	0.43	0.25				
		N	125	135	378	416	151				
	SW	tC/ha/yr	3.13								
		N	50								
	HW	tC/ha/yr	1.61	1.12	0.71	0.54	0.24				
		N	75	115	360	399	149				
Texas – South Central	All	tC/ha/yr	2.00	0.14	-0.36	-0.99					
		N	381	283	273	120					
	SW	tC/ha/yr	2.41	0.52	-0.25	-0.78					
		N	266	158	88	49					
	HW	tC/ha/yr	1.04	-0.33	-0.42	-1.14					
		N	115	125	185	71					
Utah	All	tC/ha/yr	-0.02	-0.07	0.11	0.03	-0.06		-0.18	-0.16	-0.11
		N	180	52	103	188	513		409	581	52
	SW	tC/ha/yr				0.42	-0.36		-0.80	-1.37	
		N				31	127		83	69	
	HW	tC/ha/yr				-0.53	-0.22				
		N				41	63				
	WL	tC/ha/yr	-0.06	-0.11	0.11	0.11	0.09		0.04	0.01	-0.05
		N	143	31	65	116	323		314	510	51
Vermont	All	tC/ha/yr			0.87	0.33	0.67				
		N			138	232	129				
	SW	tC/ha/yr				0.17					
		N				30					
	HW	tC/ha/yr			0.87	0.35	0.64				
		N			113	202	107				
Virginia	All	tC/ha/yr	2.78	1.28	0.92	0.63	0.90	0.97			
		N	331	261	280	401	330	33			
	SW	tC/ha/yr	3.93	1.50							
		N	171	94							
	HW	tC/ha/yr	1.55	1.16	0.96	0.66	0.94	0.97			
		N	160	167	253	377	323	33			

State	Type		0-20	21-40	41-60	61-80	81-120	121+	121-160	161-300	300+
Washington	All	tC/ha/yr	2.75	2.98	0.57	0.56	0.37		0.39	0.10	-0.25
		N	281	342	239	416	488		209	317	124
	SW	tC/ha/yr	2.74	3.06	0.89	0.56	0.42		0.40	0.10	-0.25
		N	249	321	213	375	470		208	315	124
	HW	tC/ha/yr	2.79			0.57					
		N	32			41					
Washington - Pacific Northwest - East	All	tC/ha/yr	0.74	1.49	0.90	0.33	-0.06		-0.04	-0.28	
		N	64	77	121	267	356		129	117	
	SW	tC/ha/yr	0.78	1.46	0.93	0.38	-0.01		-0.04	-0.28	
		N	62	75	118	251	343		129	116	
Washington - Pacific Northwest- West	All	tC/ha/yr	3.34	3.41	0.23	0.96	1.54		1.08	0.32	-0.34
		N	217	265	118	149	132		80	200	95
	SW	tC/ha/yr	3.39	3.55	0.83	0.92	1.60		1.13	0.31	-0.34
		N	187	246	95	124	127		79	199	95
	HW	tC/ha/yr	3.01								
		N	30								
West Virginia	All	tC/ha/yr		1.17	0.84	0.68	0.59				
		N		66	171	253	154				
	HW	tC/ha/yr		1.13	0.85	0.67	0.59				
		N		63	168	250	150				
Wisconsin	All	tC/ha/yr	1.26	0.81	0.32	0.24	0.23	0.77			
		N	326	483	828	962	458	42			
	SW	tC/ha/yr	1.61	0.76	0.27	0.31	0.39				
		N	79	123	165	113	81				
	HW	tC/ha/yr	1.15	0.82	0.33	0.23	0.19				
		N	247	360	663	849	377				
