# Supplementary material for: Aboveground live tree carbon stock and change in forests of conterminous United States: influence of stand age
Source: Carbon Balance Manag. 2023 Apr 16;18:7. doi: 10.1186/s13021-023-00227-z (PMC10108445; doi:10.1186/s13021-023-00227-z)
Supplement: Supplementary file 6 — Additional file 6: Table S6. Forested area by state, type (softwood, hardwood, woodland) and age class (kha, thousand hectares). % = percentage of total forestland in that age class. Values less than one percent are displayed as zeroes. Note that areas may not sum to total; “All” includes nonstocked forestland, while types include only forestland classified as stocked. Values are also rounded. For states that span two regions, areas are given for the entire state and the portion in each region. Values less than one percent are displayed as zeroes; empty cells indicate no data for that category. [file 13021_2023_227_MOESM6_ESM.pdf]

Table S6. Forested area by state, type (softwood, hardwood, woodland) and age class (kha, thousand hectares). % = percentage of total forestland in that age class. Values less than one percent are displayed as zeroes. Note that areas may not sum to total; “All” includes nonstocked forestland, while types include only forestland classified as stocked. Values are also rounded. For states that span two regions, areas are given for the entire state and the portion in each region. Values less than one percent are displayed as zeroes; empty cells indicate no data for that category.

State	Type		0-20	21-40	41-60	61-80	81-120	121+	121-160	161-300	300+
Alabama	All	kha	3458	2525	1565	1397	403	2			
		%	37	27	17	15	4	0			
	SW	kha	2083	1499	415	227	55				
		%	22	16	4	2	1				
	HW	kha	1319	1026	1150	1169	348	2			
		%	14	11	12	13	4	0			
Arizona	All	kha	735	183	233	582	2115		1608	1902	120
		%	10	2	3	8	28		22	25	2
	SW	kha	21	11	15	142	687		194	81	
		%	0	0	0	2	9		3	1	
	HW	kha	43	6	3	7	20		12	5	
		%	1	0	0	0	0		0	0	
	WL	kha	329	166	215	432	1408		1403	1816	120
		%	4	2	3	6	19		19	24	2
Arkansas	All	kha	1623	1292	1825	2189	728	2			
		%	21	17	24	29	10	0			
	SW	kha	988	738	469	274	115				
		%	13	10	6	4	1				
	HW	kha	578	554	1354	1911	611	2			
		%	8	7	18	25	8	0			
	WL	kha			1	4	3				
		%			0	0	0				
California	All	kha	991	689	1217	1625	3043		1273	1397	2584
		%	8	5	9	13	24		10	11	20
	SW	kha	267	402	643	1079	2233		954	1172	301
		%	2	3	5	8	17		7	9	2
	HW	kha	266	283	573	514	755		269	140	1842
		%	2	2	4	4	6		2	1	14
	WL	kha	11	4	1	31	55		50	85	440
		%	0	0	0	0	0		0	1	3

State	Type		0-20	21-40	41-60	61-80	81-120	121+	121-160	161-300	300+
Colorado	All	kha	1473	476	353	798	2663		1646	1795	79
		%	16	5	4	9	29		18	19	1
	SW	kha	227	167	112	267	1430		994	728	15
		%	2	2	1	3	15		11	8	0
	HW	kha	296	57	70	256	632		126	7	
		%	3	1	1	3	7		1	0	
	WL	kha	642	252	172	275	601		526	1060	64
		%	7	3	2	3	6		6	11	1
Connecticut	All	kha	29	30	55	232	369	10			
		%	4	4	8	32	51	1			
	SW	kha	3	0	1	9	14				
		%	0	0	0	1	2				
	HW	kha	16	30	54	222	354	10			
		%	2	4	7	31	49	1			
Delaware	All	kha	12	18	19	42	52	1			
		%	8	13	13	29	36	1			
	SW	kha	5	8	5	6	4				
		%	3	5	4	4	3				
	HW	kha	6	10	14	36	48	1			
		%	4	7	10	25	33	1			
Florida	All	kha	1902	1785	1350	1113	653	65			
		%	28	26	20	16	10	1			
	SW	kha	1057	1049	470	302	117	4			
		%	15	15	7	4	2	0			
	HW	kha	613	735	880	810	536	62			
		%	9	11	13	12	8	1			
Georgia	All	kha	3211	2963	1480	1466	751	29			
		%	32	30	15	15	8	0			
	SW	kha	1733	1893	473	311	70	5			
		%	18	19	5	3	1	0			
	HW	kha	1342	1071	1007	1155	681	24			
		%	14	11	10	12	7	0			

State	Type		0-20	21-40	41-60	61-80	81-120	121+	121-160	161-300	300+
Idaho	All	kha	2132	526	600	1155	2436		1161	697	79
		%	24	6	7	13	28		13	8	1
	SW	kha	1130	461	536	1059	2303		1112	656	79
		%	13	5	6	12	26		13	7	1
	HW	kha	148	45	36	54	63		2		
		%	2	1	0	1	1		0		
	WL	kha	12	20	28	42	70		47	41	
		%	0	0	0	0	1		1	0	
Illinois	All	kha	116	210	539	566	473	60			
		%	6	11	27	29	24	3			
	SW	kha	4	5	10	11	1				
		%	0	0	1	1	0				
	HW	kha	98	205	529	554	473	60			
		%	5	10	27	28	24	3			
Indiana	All	kha	87	202	518	605	497	23			
		%	5	10	27	31	26	1			
	SW	kha	4	9	15	13	5				
		%	0	0	1	1	0				
	HW	kha	74	192	503	592	492	23			
		%	4	10	26	31	25	1			
Iowa	All	kha	57	163	330	351	223	40			
		%	5	14	28	30	19	3			
	SW	kha	1	8	5	1					
		%	0	1	0	0					
	HW	kha	38	155	324	350	223	40			
		%	3	13	28	30	19	3			
Kansas	All	kha	118	279	386	159	63				
		%	12	28	38	16	6				
	SW	kha	4	21	15	4	3				
		%	0	2	1	0	0				
	HW	kha	88	258	371	155	60				
		%	9	26	37	15	6				

State	Type		0-20	21-40	41-60	61-80	81-120	121+	121-160	161-300	300+
Kentucky	All	kha	272	572	1759	1867	538	1			
		%	5	11	35	37	11	0			
	SW	kha	24	70	58	23	3				
		%	0	1	1	0	0				
	HW	kha	235	502	1701	1844	535	1			
		%	5	10	34	37	11	0			
Louisiana	All	kha	2146	1507	1106	1029	281	2			
		%	35	25	18	17	5	0			
	SW	kha	1200	867	227	151	16				
		%	20	14	4	2	0				
	HW	kha	877	640	880	878	264	2			
		%	14	11	14	14	4	0			
Maine	All	kha	286	1669	1569	1704	1631	223			
		%	4	24	22	24	23	3			
	SW	kha	121	819	492	599	741	162			
		%	2	12	7	8	10	2			
	HW	kha	148	850	1077	1105	890	61			
		%	2	12	15	16	13	1			
Maryland	All	kha	85	93	165	238	373	35			
		%	9	9	17	24	38	4			
	SW	kha	47	42	39	21	16	3			
		%	5	4	4	2	2	0			
	HW	kha	32	50	126	217	357	32			
		%	3	5	13	22	36	3			
Massachusetts	All	kha	18	36	134	457	562	11			
		%	1	3	11	38	46	1			
	SW	kha		9	22	63	110	6			
		%		1	2	5	9	0			
	HW	kha	12	27	112	394	452	5			
		%	1	2	9	32	37	0			
Michigan	All	kha	734	972	1713	2486	2117	139			
		%	9	12	21	30	26	2			
	SW	kha	182	286	450	533	496	71			
		%	2	4	6	7	6	1			
	HW	kha	483	687	1263	1953	1621	67			
		%	6	8	15	24	20	1			

State	Type		0-20	21-40	41-60	61-80	81-120	121+	121-160	161-300	300+
Minnesota	All	kha	1217	1270	1340	1604	1436	285			
		%	17	18	19	22	20	4			
	SW	kha	190	323	432	477	552	210			
		%	3	5	6	7	8	3			
	HW	kha	948	948	908	1127	885	75			
		%	13	13	13	16	12	1			
Mississippi	All	kha	2336	2597	1401	1223	215				
		%	30	33	18	16	3				
	SW	kha	1369	1618	385	217	40				
		%	18	21	5	3	1				
	HW	kha	822	980	1016	1006	175				
		%	11	13	13	13	2				
Missouri	All	kha	186	563	1515	2235	1586	101			
		%	3	9	24	36	26	2			
	SW	kha	16	57	83	69	31	1			
		%	0	1	1	1	0	0			
	HW	kha	144	506	1432	2166	1556	100			
		%	2	8	23	35	25	2			
Montana	All	kha	2379	706	619	861	2950		1601	1291	63
		%	23	7	6	8	28		15	12	1
	SW	kha	1335	617	503	704	2720		1528	1251	60
		%	13	6	5	7	26		15	12	1
	HW	kha	149	32	23	37	68		10	6	
		%	1	0	0	0	1		0	0	
	WL	kha	44	58	92	120	162		63	34	2
		%	0	1	1	1	2		1	0	0
Nebraska	All	kha	83	95	126	135	128		1		
		%	15	17	22	24	23		0		
	SW	kha	12	28	43	33	49				
		%	2	5	8	6	9				
	HW	kha	26	67	81	102	76		1		
		%	5	12	14	18	13		0		
	WL	kha			2		2				
		%			0		0				

State	Type		0-20	21-40	41-60	61-80	81-120	121+	121-160	161-300	300+
Nevada	All	kha	438	84	154	333	922		1026	1225	111
		%	10	2	4	8	21		24	29	3
	SW	kha	3	2	7	6	34		41	62	14
		%	0	0	0	0	1		1	1	0
	HW	kha	40	18	10	17	12		3		
		%	1	0	0	0	0		0		
	WL	kha	94	63	136	310	876		982	1163	97
		%	2	1	3	7	20		23	27	2
New Hampshire	All	kha	85	110	321	770	596	16			
		%	4	6	17	41	31	1			
	SW	kha	14	30	44	174	156	4			
		%	1	2	2	9	8	0			
	HW	kha	65	80	277	596	440	12			
		%	3	4	15	31	23	1			
New Jersey	All	kha	38	46	163	248	263	44			
		%	5	6	20	31	33	6			
	SW	kha	12	17	52	66	47	6			
		%	1	2	6	8	6	1			
	HW	kha	16	29	111	182	216	38			
		%	2	4	14	23	27	5			
New Mexico	All	kha	1687	567	534	970	2792		1856	1523	36
		%	17	6	5	10	28		19	15	0
	SW	kha	23	26	33	199	917		408	195	
		%	0	0	0	2	9		4	2	
	HW	kha	49	4	9	46	86		17	3	
		%	0	0	0	0	1		0	0	
	WL	kha	972	537	492	725	1790		1431	1325	36
		%	10	5	5	7	18		14	13	0
New York	All	kha	245	603	1635	2511	2340	201			
		%	3	8	22	33	31	3			
	SW	kha	10	57	216	300	253	48			
		%	0	1	3	4	3	1			
	HW	kha	152	546	1420	2212	2087	153			
		%	2	7	19	29	28	2			

State	Type		0-20	21-40	41-60	61-80	81-120	121+	121-160	161-300	300+
North Carolina	All	kha	2026	1682	1292	1458	1086	44			
		%	27	22	17	19	14	1			
	SW	kha	983	977	397	203	78	5			
		%	13	13	5	3	1	0			
	HW	kha	974	705	895	1254	1009	39			
		%	13	9	12	17	13	1			
North Dakota	All	kha	25	48	88	88	77		1		
		%	7	15	27	27	24		0		
	SW	kha					2				
		%					1				
	HW	kha	17	47	80	69	61		1		
		%	5	14	25	21	19		0		
	WL	kha		1	8	19	14				
		%		0	2	6	4				
Ohio	All	kha	251	335	979	1012	570	34			
		%	8	11	31	32	18	1			
	SW	kha	21	7	31	22	2				
		%	1	0	1	1	0				
	HW	kha	203	329	948	990	568	34			
		%	6	10	30	31	18	1			
Oklahoma	All	kha	1993	2410	2646	2197	422				
		%	21	25	27	23	4				
	SW	kha	448	532	268	140	37				
		%	5	6	3	1	0				
	HW	kha	1090	1717	2266	2048	385				
		%	11	18	23	21	4				
	WL	kha	114	161	111	10					
		%	1	2	1	0					
Oklahoma - GP	All	kha	1161	1403	1484	964	137				
		%	12	15	15	10	1				
	SW	kha	89	182	90	18					
		%	1	2	1	0					
	HW	kha	685	1060	1283	937	137				
		%	7	11	13	10	1				
	WL	kha	114	161	111	10					
		%	1	2	1	0					

State	Type		0-20	21-40	41-60	61-80	81-120	121+	121-160	161-300	300+
Oklahoma – SC	All	kha	832	1007	1162	1232	284				
		%	9	10	12	13	3				
	SW	kha	359	350	178	121	37				
		%	4	4	2	1	0				
	HW	kha	405	657	984	1111	248				
		%	4	7	10	11	3				
Oregon	All	kha	1693	1831	1693	1605	2700		1048	1057	338
		%	14	15	14	13	23		9	9	3
	SW	kha	1000	1623	1431	1446	2486		1004	1033	272
		%	8	14	12	12	21		8	9	2
	HW	kha	215	207	259	159	208		44	24	65
		%	2	2	2	1	2		0	0	1
	WL	kha	1	1	3		6		0		
		%	0	0	0		0		0		
Oregon - PWE	All	kha	585	526	610	1031	1927		578	472	70
		%	5	4	5	9	16		5	4	1
	SW	kha	240	518	599	1017	1879		572	472	51
		%	2	4	5	9	16		5	4	0
	HW	kha	17	7	8	14	42		6	0	19
		%	0	0	0	0	0		0	0	0
	WL	kha	1	1	3		6		0		
		%	0	0	0		0		0		
Oregon - PWW	All	kha	1108	1305	1084	574	773		470	586	268
		%	9	11	9	5	6		4	5	2
	SW	kha	759	1105	832	429	607		432	562	221
		%	6	9	7	4	5		4	5	2
	HW	kha	198	200	252	145	166		38	24	46
		%	2	2	2	1	1		0	0	0
Pennsylvania	All	kha	300	524	1100	1746	2963	147			
		%	4	8	16	26	44	2			
	SW	kha	8	30	62	68	85	6			
		%	0	0	1	1	1	0			
	HW	kha	247	493	1038	1678	2879	141			
		%	4	7	15	25	42	2			

State	Type		0-20	21-40	41-60	61-80	81-120	121+	121-160	161-300	300+
Rhode Island	All	kha	2	6	17	73	51				
		%	1	4	11	49	34				
	SW	kha		2	1	9	5				
		%		1	1	6	4				
	HW	kha	0	4	15	64	46				
		%	0	3	10	43	31				
South Carolina	All	kha	1669	1711	772	697	344	9			
		%	32	33	15	13	7	0			
	SW	kha	850	1162	282	127	67				
		%	16	22	5	2	1				
	HW	kha	763	550	490	570	277	9			
		%	15	11	9	11	5	0			
South Dakota	All	kha	121	50	108	149	269		62	9	
		%	16	6	14	19	35		8	1	
	SW	kha	25	34	58	87	206		54	9	
		%	3	4	8	11	27		7	1	
	HW	kha	15	14	45	53	55		7		
		%	2	2	6	7	7		1		
	WL	kha	2	2	6	9	9		1		
		%	0	0	1	1	1		0		
Tennessee	All	kha	762	631	1100	1926	1159	39			
		%	14	11	20	34	21	1			
	SW	kha	228	168	91	85	23				
		%	4	3	2	2	0				
	HW	kha	519	463	1010	1842	1137	39			
		%	9	8	18	33	20	1			
Texas	All	kha	12788	14588	14511	6770	1370		112	25	6
		%	25	29	29	13	3		0	0	0
	SW	kha	1897	1644	714	365	84				
		%	4	3	1	1	0				
	HW	kha	2525	3744	5727	3369	762		82	25	
		%	5	7	11	7	2		0	0	
	WL	kha	5014	9200	8070	3035	524		30		6
		%	10	18	16	6	1		0		0

State	Type		0-20	21-40	41-60	61-80	81-120	121+	121-160	161-300	300+
Texas – GP	All	kha	9282	11697	12369	5640	1256		112	25	6
		%	19	23	25	11	3		0	0	0
	SW	kha	7	70	28	56	29				
		%	0	0	0	0	0				
	HW	kha	1044	2414	4271	2548	702		82	25	
		%	2	5	9	5	1		0	0	
	WL	kha	5006	9195	8070	3035	524		30		6
		%	10	18	16	6	1		0		0
Texas - SC	All	kha	3507	2909	2142	1130	114				
		%	7	6	4	2	0				
	SW	kha	1890	1574	686	309	54				
		%	4	3	1	1	0				
	HW	kha	1481	1330	1456	821	60				
		%	3	3	3	2	0				
	WL	kha	8	5							
		%	0	0							
Utah	All	kha	1017	289	233	587	1626		1300	2062	172
		%	14	4	3	8	22		18	28	2
	SW	kha	81	90	32	85	389		263	216	
		%	1	1	0	1	5		4	3	
	HW	kha	126	64	67	132	222		44	2	
		%	2	1	1	2	3		1	0	
	WL	kha	542	135	134	369	1015		993	1843	172
		%	7	2	2	5	14		14	25	2
Vermont	All	kha	47	114	308	703	650	8			
		%	3	6	17	38	36	0			
	SW	kha	13	15	51	121	89	5			
		%	1	1	3	7	5	0			
	HW	kha	32	99	258	582	561	3			
		%	2	5	14	32	31	0			
Virginia	All	kha	1267	1183	1016	1198	1690	148			
		%	19	18	16	18	26	2			
	SW	kha	548	493	163	85	62				
		%	8	8	3	1	1				
	HW	kha	685	690	853	1113	1628	148			
		%	11	11	13	17	25	2			

State	Type		0-20	21-40	41-60	61-80	81-120	121+	121-160	161-300	300+
Washington	All	kha	1500	1513	945	1253	1810		660	845	419
		%	17	17	11	14	20		7	9	5
	SW	kha	901	1309	789	1070	1689		647	841	408
		%	10	15	9	12	19		7	9	5
	HW	kha	192	204	156	183	121		13	4	10
		%	2	2	2	2	1		0	0	0
Washington - PWE	All	kha	576	290	369	712	1295		426	332	77
		%	6	3	4	8	14		5	4	1
	SW	kha	251	277	356	657	1234		417	329	69
		%	3	3	4	7	14		5	4	1
	HW	kha	22	13	13	56	61		9	3	7
		%	0	0	0	1	1		0	0	0
Washington - PWW	All	kha	924	1223	577	541	515		234	513	342
		%	10	14	6	6	6		3	6	4
	SW	kha	650	1032	434	413	455		229	512	339
		%	7	12	5	5	5		3	6	4
	HW	kha	170	191	143	127	60		5	1	3
		%	2	2	2	1	1		0	0	0
West Virginia	All	kha	214	264	951	1721	1613	87			
		%	4	5	20	35	33	2			
	SW	kha	19	10	33	36	22	3			
		%	0	0	1	1	0	0			
	HW	kha	183	253	919	1685	1591	84			
		%	4	5	19	35	33	2			
Wisconsin	All	kha	753	976	1524	1887	1613	111			
		%	11	14	22	27	24	2			
	SW	kha	114	269	339	285	223	54			
		%	2	4	5	4	3	1			
	HW	kha	575	707	1185	1602	1390	57			
		%	8	10	17	23	20	1			

State	Type		0-20	21-40	41-60	61-80	81-120	121+	121-160	161-300	300+
Wyoming	All	kha	1106	260	118	268	1151		678	633	25
		%	26	6	3	6	27		16	15	1
	SW	kha	550	208	83	177	975		586	517	14
		%	13	5	2	4	23		14	12	0
	HW	kha	160	34	16	47	85		15		
		%	4	1	0	1	2		0		
	WL	kha	25	18	19	44	91		77	116	10
		%	1	0	0	1	2		2	3	0
